# Biomarkers of severity and threshold of allergic reactions during oral peanut challenges

**DOI:** 10.1016/j.jaci.2020.03.035

**Published:** 2020-08

**Authors:** Alexandra F. Santos, George Du Toit, Colin O’Rourke, Natalia Becares, Natália Couto-Francisco, Suzana Radulovic, Ekaterina Khaleva, Monica Basting, Kristina M. Harris, David Larson, Peter Sayre, Marshall Plaut, Graham Roberts, Henry T. Bahnson, Gideon Lack

**Affiliations:** a Department of Women and Children’s Health (Pediatric Allergy), School of Life Course Sciences, Faculty of Life Sciences and Medicine, London, United Kingdom; b Peter Gorer Department of Immunobiology, School of Immunology and Microbial Sciences, King’s College London, London, United Kingdom; c Children’s Allergy Service, Evelina London Children's Hospital, Guy’s and St Thomas’ Hospital, London, United Kingdom; d Asthma UK Centre in Allergic Mechanisms of Asthma, London, United Kingdom; e Immune Tolerance Network, Benaroya Research Institute, Seattle, Wash; f Immune Tolerance Network, Bethesda, Md; g Division of Hematology-Oncology, Department of Medicine, University of California, San Francisco, Calif; h National Institute of Allergy and Infectious Diseases, Bethesda, Md; i David Hide Asthma and Allergy Research Centre, St Mary’s Hospital, Isle of Wight, Southampton, United Kingdom; j National Institute for Health Research Biomedical Research Centre, University Hospital Southampton NHS Foundation Trust and Clinical and Experimental Sciences Academic Unit, University of Southampton Faculty of Medicine, Southampton, United Kingdom

**Keywords:** Basophil, basophil activation test, diagnosis, food allergy, LEAP study, peanut allergy, severity, threshold, adverse events, Ara h, *Arachis hypogaea*, BAT, Basophil activation test, CD-sens, Allergen threshold sensitivity, fMLP, Formyl-methionyl-leucylphenylalanine, LEAP, Learning Early about Peanut Allergy, LEAP-On, Persistence of Oral Tolerance to Peanut, OFC, Oral food challenge, PA, Peanut allergy, PAS, Peanut Allergy Sensitization, rAra h, Recombinant *Arachis hypogaea*, SPT, Skin prick test

## Abstract

**Background:**

Oral food challenge (OFC) is the criterion standard to assess peanut allergy (PA), but it involves a risk of allergic reactions of unpredictable severity.

**Objective:**

Our aim was to identify biomarkers for risk of severe reactions or low dose threshold during OFC to peanut.

**Methods:**

We assessed Learning Early about Peanut Allergy study, Persistance of Oral Tolerance to Peanut study, and Peanut Allergy Sensitization study participants by administering the basophil activation test (BAT) and the skin prick test (SPT) and measuring the levels of peanut-specific IgE, *Arachis hypogaea* 2–specific IgE, and peanut-specific IgG4, and we analyzed the utility of the different biomarkers in relation to PA status, severity, and threshold dose of allergic reactions to peanut during OFC.

**Results:**

When a previously defined optimal cutoff was used, the BAT diagnosed PA with 98% specificity and 75% sensitivity. The BAT identified severe reactions with 97% specificity and 100% sensitivity. The SPT, level of *Arachis hypogaea* 2–specific IgE, level of peanut-specific IgE, and IgG4/IgE ratio also had 100% sensitivity but slightly lower specificity (92%, 93%, 90%, and 88%, respectively) to predict severity. Participants with lower thresholds of reactivity had higher basophil activation to peanut *in vitro*. The SPT and the BAT were the best individual predictors of threshold. Multivariate models were superior to individual biomarkers and were used to generate nomograms to calculate the probability of serious adverse events during OFC for individual patients.

**Conclusions:**

The BAT diagnosed PA with high specificity and identified severe reactors and low threshold with high specificity and high sensitivity. The BAT was the best biomarker for severity, surpassed only by the SPT in predicting threshold. Nomograms can help estimate the likelihood of severe reactions and reactions to a low dose of allergen in individual patients with PA.

## Graphical abstract

**Figure:**
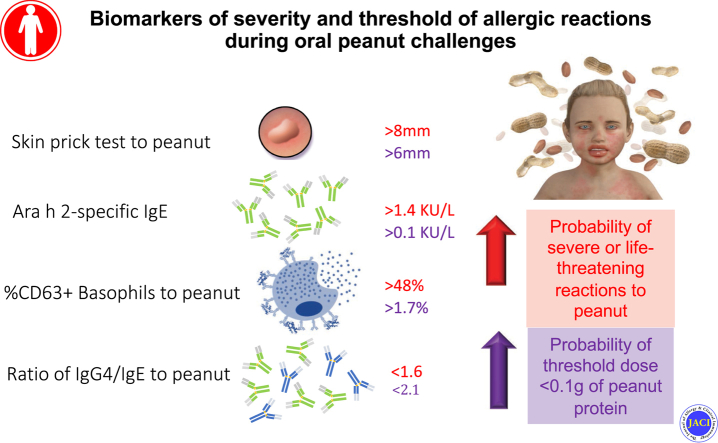

Oral food challenge (OFC) is the criterion standard to diagnose and assess resolution of food allergies. OFC is also the criterion standard to confirm the eligibility of patients with food allergy for clinical trials of experimental treatments for food allergy and to assess patients’ clinical responses to such treatments. OFC can result in potentially severe allergic reactions and requires an experienced and highly skilled clinical team with the ability and equipment needed to treat anaphylaxis. Although rare, 2 deaths have been reported in children undergoing OFC.^1^^,^^2^ A biomarker that could identify individuals at high-risk for severe allergic reactions and/or for reacting to small amounts of the allergens would ensure patients’ safety and comfort and improve current management of patients with food allergy.

Peanut is one of the main culprits of fatal and near-fatal food-induced allergic reactions.^3^ The prevalence of peanut allergy (PA) is increasing, and some studies have reported an increase in food-induced anaphylaxis fatalities.^4^ Evidence about the utility of the skin prick test (SPT) and specific IgE level to predict the severity of allergic reactions to peanut is conflicting.^5^, ^6^, ^7^, ^8^ *Arachis hypogaea* 2 (Ara h 2)-specific IgE is a discriminative marker to diagnose PA, and an association with severity has been observed.^9^^,^^10^ The basophil activation test (BAT) has shown the highest accuracy for diagnosing PA, with a higher proportion of activated basophils (as measured by percentage of CD63^+^ basophils) being associated with more severe allergic reactions during OFC^9^^,^^11^^,^^12^ and the cumulative threshold dose being associated with the concentration at which the basophils reacted *in vitro.*^9^^,^^13^ Despite the need for fresh blood samples and the existence of a subset of individuals with nonresponder basophils, the BAT has emerged as a promising biomarker to identify high-risk patients with PA.

The Learning Early about Peanut Allergy (LEAP) study^14^ conferred a unique opportunity to perform the BAT and other tests in a large number of well-characterized children who were being assessed for PA. We aimed to assess their utility in diagnosing PA and in predicting severity and threshold dose of allergic reactions during oral peanut challenges in this and related cohorts.

## Methods

### Study cohorts

Participants in the LEAP,^15^ Persistence of Oral Tolerance to Peanut (LEAP-On),^16^ and PA Sensitization (PAS) studies were included (see Table E1 in this article’s Online Repository at www.jacionline.org). The LEAP study was a large interventional study that assessed the effect of early peanut consumption on the development of PA by age 5 years.^15^ The LEAP study participants (groups II and III of the LEAP screening study^14^) were followed up after 1 year of peanut avoidance in the LEAP-On study.^16^ Children who had been excluded from the LEAP study as infants were included in the PAS study.^14^ An additional cohort of children recruited from specialized paediatric allergy clinics in London^9^ was included for external validation (see Table E2 in this article’s Online Repository at www.jacionline.org). All studies were approved by the relevant Research Ethics Committees in the United Kingdom (reference numbers 04/Q0403/13 [LEAP study], 10/H0711/77 [LEAP-On study], 11/LO/0045 [PAS study], and 10/H0802/044 [clinic cohort]).

We aimed to perform the BAT in LEAP study participants who showed evidence of peanut sensitization at any time point in the LEAP study on either the SPT (≥1 mm) or serum level of specific IgE (≥0.10 kU/L) to peanut and in 50 LEAP study participants who showed a peanut SPT result of 0 mm and a specific level of IgE to peanut less than 0.1 kU/L at all time points of the study before their assessment. We aimed to repeat the BAT to peanut at the end of the LEAP-On study in the same participants. In the PAS study, we aimed to test all participants who were assessed at approximately 60 months of age.^14^

### Skin prick testing and determination of IgE and IgG4 levels

The SPT and BAT were performed on the same day as OFC. Peanut-specific IgE and IgG4 levels were determined by using samples collected when blood was collected for the BAT. Skin prick testing was performed by using a commercially available peanut extract (ALK-Abelló, Hørsholm, Denmark), as previously described.^15^ The levels of specific IgE to peanut and the peanut allergens recombinant *Arachis hypogaea* (rAra h) 1, rAra h 2, rAra h 3, rAra h 8, and rAra h 9 and the levels of peanut-specific IgG4 in the serum were measured by using an immunoenzymatic assay (ImmunoCAP, ThermoFisher, Uppsala, Sweden).

### BAT

**Fig E1:**
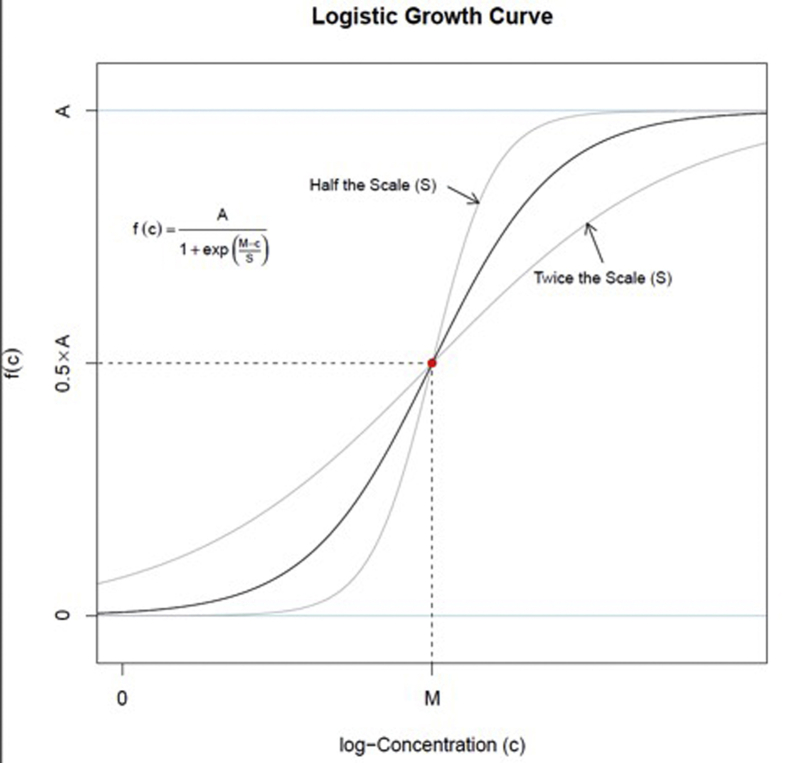


Whole blood was collected into lithium heparin tubes and used to perform the BAT to peanut extract on the same day and within 4 hours of blood collection, as previously described.^11^ Briefly, 100 μL of whole blood was incubated with the same volume of peanut extract (ALK-Abelló) diluted in RPMI medium (GIBCO, Paisley, United Kingdom), anti-IgE (1 μg/ml, Sigma-Aldrich, Poole, United Kingdom), anti-FceRI (2.5 μg/mL, eBioscience, San Diego, Calif), formyl-methionyl-leucylphenylalanine (fMLP) (1 μM [Sigma-Aldrich]), or RPMI medium alone. Cells were stained with CD123-FITC (eBioscience, San Diego, Calif), CD203c-PE, HLA-DR-PerCP, and CD63-allophycocyanin (Biolegend, San Diego, Calif). Flow cytometry was performed by using FACS CantoII with FACSDiva software (BD Biosciences, San Jose, Calif). A mininum of 500 basophils were acquired. The flow cytometry data were analyzed by using FlowJo software (version 7.6.1 [TreeStar, Ashland, Ore]). Basophils were gated as SSC^low^/CD203c^+^/CD123^+^/HLA-DR^–^.^11^^,^^17^ Basophil activation was expressed as percentage of CD63^+^ basophils. Individuals with nonresponder basophils were defined as having a percentage of CD63^+^ basophils less than 5% to both IgE-mediated positive controls (anti-IgE and anti-FcεRI) and were excluded from statistical analyses. Allergen threshold sensitivity (CD-sens) was calculated for each BAT by estimating the parameters of a logistic growth function, one of which is the reciprocal of CD-sens, by using a nonlinear model (see Fig E1 in this article’s Online Repository at www.jacionline.org).

### Oral peanut challenges

Oral peanut challenges were performed according to the LEAP study protocol as previously reported.^15^^,^^16^ Participants who were not IgE-sensitized to peanut and had no history of reaction to peanut, no diagnosis of or suspected allergy to sesame or tree nuts, and no history of anaphylaxis underwent open challenge with a cumulative dose of up to 5 g of peanut protein given as a single dose. Open challenge is the criterion standard to confirm peanut tolerance, and all open challenges were indeed negative for PA. All other participants underwent a double-blind, placebo-controlled peanut challenge to a cumulative dose of up to 9.35 g of peanut protein given as incremental doses (see Table E3 in this article’s Online Repository at www.jacionline.org). Participants with suspected PA, a history of life-threatening food-induced anaphylaxis, or an SPT result of 7 mm or larger received an additional lower starting active dose of 0.033 g of peanut protein. Some doses may have been repeated at the discretion of the investigator performing OFC. The challenge was considered positive only when objective signs of an allergic reaction developed (see Table E4 in this article’s Online Repository at www.jacionline.org), and the symptoms were treated according to local guidelines, which follow the UK Resuscitation Council recommendation to administer epinephrine to patients with life-threatening airway (swelling, hoarseness, stridor) and/or breathing (tachypnoea, wheeze, fatigue, cyanosis, specific oxygen saturation as measured by pulse oximetry reading less than 90%, confusion) and/or circulation (pale, clammy, hypotension, faintness, drowsiness, coma) problems.^18^ For participants for whom the outcome of OFC was inconclusive or not available, PA was assessed by a diagnostic algorithm using the SPT and level of specific IgE to peanut. The procedure for OFC in the cohort used for external validation was similar to that already described for the majority of study subjects and was previously published.^11^

The severity of allergic reactions during OFC were classified according to different severity scores represented in Table E5 (available in this article’s Online Repository at www.jacionline.org).^19^^,^^20^ The threshold dose was determined as the cumulative amount of peanut protein (in grams) tolerated during OFC. The cumulative tolerated dose was set to 9.35 g for all participants who passed OFC.

### Statistical analyses

Continuous variables were summarized by quantiles, and categorical variables were summarized by counts and percentages. Comparisons of continuous predictors use either the Student *t*-test or the Wilcoxon rank sum-test. Receiver operator characteristic curve analyses were performed to assess the diagnostic performance of each individual biomarker to predict severe reactions. The performance of the optimal cutoffs was described by using sensitivity, specificity, positive predictive value, and negative predictive value.

CD-sens, the reciprocal of median effective dose, was estimated by fitting a hierarchical Bayesian nonlinear model (see Fig E1) to the BAT curve for each sample. This model assumes that each BAT curve follows the form of a 3-parameter logistic growth curve. The resulting logistic curve midpoint parameter estimates from this model were used to estimate CD-sens. Model fitting was performed by using the Markov chain Monte Carlo method, which was performed by using the Stan software.^21^

Proportional odds logistic regression models were created for prediction of reaction severity. The predictors used in these models were chosen on the basis of being established predictors of reaction to peanut, and no further variable selection was done on the basis of tests of statistical hypotheses. Model performance was described by using bootstrap bias-corrected concordance probabilities (C-statistics) and calibration accuracy measurements (mean absolute prediction error and 90th percentile of absolute prediction error). External model validation was done by using data available from participants in a separate clinic cohort (see Table E2). Statistical analysis was done in R software (version 3.5.2) and JMP Pro (version 14). Data sets are available through TrialShare, a public website managed by the Immune Tolerance Network (https://www.itntrialshare.org/LEAP_JACI_BAT.url).

## Results

### Study population

**Fig E2:**
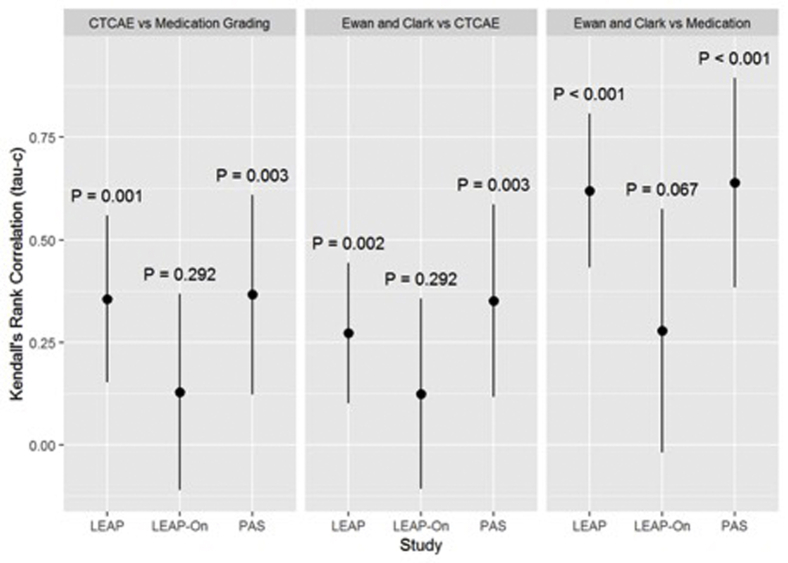


There were 468 individual subjects enrolled across the LEAP, LEAP-On, and PAS studies, and from these the BAT was performed in 706 blood samples (335 from LEAP study participants, 295 from LEAP-On study participants, and 76 from PAS study participants) on the day of the study visits at which allergic status to peanut was assessed (Table I^18^ and see Table E6 in this article’s Online Repository at www.jacionline.org). An additional 123 subjects were eligible for the BAT but were not tested for logistic reasons (see Tables E7 and E8 in this article’s Online Repository at www.jacionline.org). On the BAT, in 93 samples (13.1%) basophils did not react to the IgE-mediated positive controls and were excluded from statistical analyses. The excluded samples included those from 11 subjects who were allergic to peanut (10.8% of nonresponders had PA, and 6.7% of those with PA were nonresponders); also excluded were 5 samples whose response status could not be determined on account of missing data. Subjects with undetectable peanut-specific IgE (<0.10 kU/L) who were not tested on the BAT (n = 373) were imputed to have a BAT result of 0 because all patients with undetectable IgE had negative a BAT result in both this and previous studies.^11^ A total of 981 cases with performed or imputed BAT results were available for building predictive models, and 558 independent samples from the LEAP and PAS studies were used for all other analyses. In all, 14% percent of patients were allergic to peanut. The threshold cumulative dose ranged between 0 and 10.1 g of peanut protein (between 5 and 10.1 g in participants without allergy and between 0 and 4.5 g in participants with allergy, respectively). The cumulative dose exceeded 9.35 g in participants who were given repeat doses at the discretion of the investigators. According to the Common Terminology Criteria for Adverse Events scale, 15%, 3%, and 16% participants in the LEAP, LEAP-On, and PAS studies had severe reactions, respectively. There was some degree of concordance with other severity scales (Kendall τ = 0.12-0.64 [see Fig E2 in this article’s Online Repository at www.jacionline.org]).

**Table I:** Demographic and clinical characteristics of participants in the LEAP, LEAP-On, and PAS studies included in the analyses

Characteristic	LEAP			LEAP-On			PAS			
	Peanut avoid	Peanut consume	Overall	Peanut avoid	Peanut consume	Overall	Group I	Group IV	Group V	Overall
	(n = 252)	(n = 222)	(n = 474)	(n = 219)	(n = 204)	(n = 423)	(n = 36)	(n = 45)	(n = 3)	(n = 84)
Male sex	164 (65%)	119 (54%)	283 (60%)	136 (62%)	112 (55%)	248 (59%)	24 (67%)	31 (69%)	1 (33%)	56 (67%)
Atopic eczema∗	104 (41%)	80 (36%)	184 (39%)	81 (37%)	79 (39%)	160 (38%)	32/35 (91%)	44/44 (100%)	2 (67%)	78/82 (95%)
Participants with PA diagnosed	46/250 (18%)	8 (4%)	54/472 (11%)	42 (19%)	9/203 (4%)	51/422 (12%)	0 (0%)	36/44 (82%)	1 (33%)	33/79 (42%)
Participants with positive OFC to peanut	42/248 (17%)	7/221 (3%)	49/469 (10%)	26/202 (13%)	7/200 (4%)	33/402 (8%)	0 (0%)	34/42 (81%)	1 (33%)	35/81 (43%)
Ewan & Clark grade severe	12/41 (29%)	2/7 (29%)	14/48 (29%)	1/25 (4%)	1/7 (14%)	2/32 (6%)	(n = 0)	8/32 (25%)	0/1 (0%)	8/33 (24%)
Medication grade severe	7/41 (17%)	2/7 (29%)	9/48 (19%)	1/25 (4%)	0/7 (0%)	1/32 (3%)	(n = 0)	7/32 (22%)	0/1 (0%)	7/33 (21%)
CTCAE grade severe	7/41 (17%)	0/7 (0%)	7/48 (15%)	1/24 (4%)	0/7 (0%)	1/31 (3%)	(n = 0)	5/31 (16%)	0/1 (0%)	5/32 (16%)
Threshold dose of reaction during OFC (g)†	0.1 [0.0-0.8] (n = 42)	0.1 [0.0-0.1] (n = 7)	0.1 [0.0-0.8] (n = 49)	0.8 [0.0-4.3] (n = 26)	1.2 [0.4-3.1] (n = 7)	0.8 [0.0-4.3] (n = 33)	(n = 0)	0.0 [0.0-0.1] (n = 34)	0.4 [0.4-0.4] (n = 1)	0.0 [0.0-0.2] (n = 35)

*CTCAE*, Common Terminology Criteria for Adverse Events; *OFC*, oral food challenge.^18^

∗ Eczema based on SCORAD score of more than 0.

† Median [interquartile range].

### The BAT confirmed its high specificity to diagnose PA

**Fig 1:**
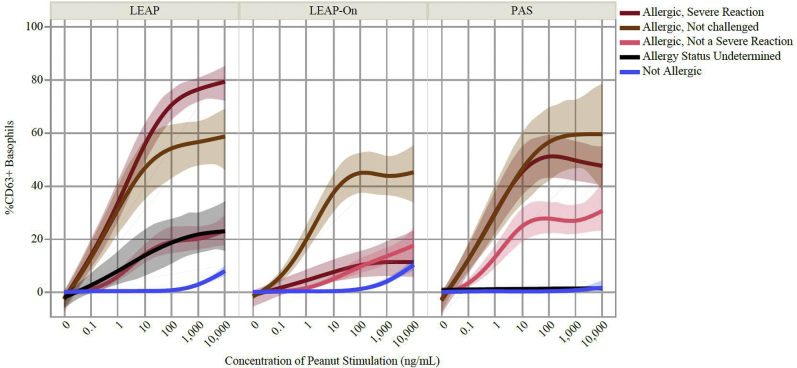
Mean basophil activation measured as percentage of CD63-positive (%CD63+) basophils corrected for spontaneous activation (ie, minus %CD63^+^ basophils in the absence of *in vitro* stimulation) in response to increasing concentrations of peanut extract in LEAP, LEAP-On, and PAS study participants by allergic status. The BAT and allergic status were determined when subjects were approximately 5 years of age in the LEAP and PAS studies and approximately 6 years of age in the LEAP-On study. Each regression line represents a smoothed mean dose response curve using a cubic spline with 95% bootstrapped CIs for each combination of allergic status and reaction severity group.

**Fig E3:**
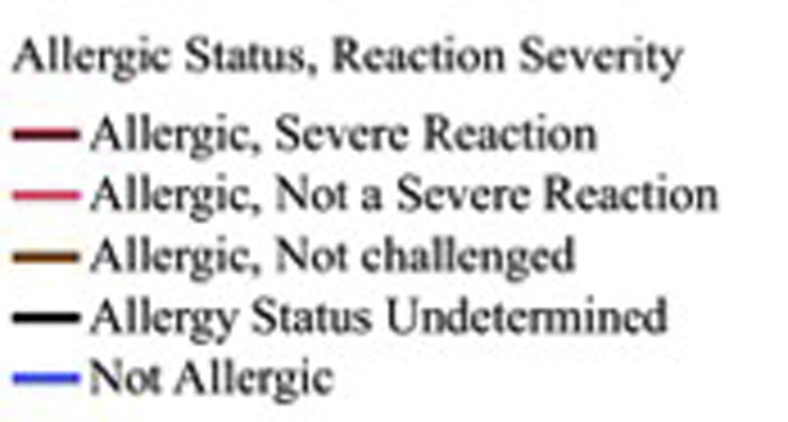


**Fig E4:**
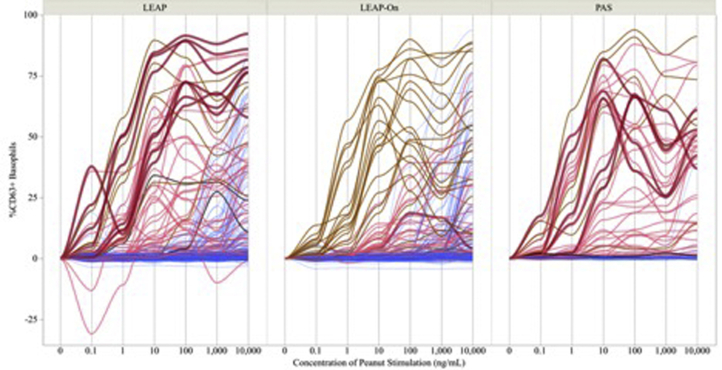


Basophil activation to peanut was higher in subjects with PA than in subjects without PA (Fig 1 and see Fig E3 in this article’s Online Repository at www.jacionline.org), with risk of reaction at the 75th percentile of the BAT results being 3.85-fold higher than the 25th percentile of the BAT results (hazard ratio = 3.85; 95% CI, 3.2-4.7; *P* < .001). Using data from participants in the LEAP and PAS studies, we applied the optimal diagnostic cutoff previously identified for BAT to peanut^11^ (ie, 4.78% CD63^+^ basophils for average BAT result at 10 and 100 ng/mL of peanut extract), which had 74.7% sensitivity, 98.7% specificity, a 95.4% negative predictive value, a 91.5% positive predictive value, and 57.8 and 0.3 positive and negative likelihood ratios, respectively, to diagnose PA. Basophil activation in response to anti-IgE, anti-FcεRI, and fMLP was similar across allergic status (see Fig E4 in this article’s Online Repository at www.jacionline.org).

### The BAT and other tests identified participants with severe or life-threatening allergic reactions during oral peanut challenges with high accuracy

**Fig 2:**
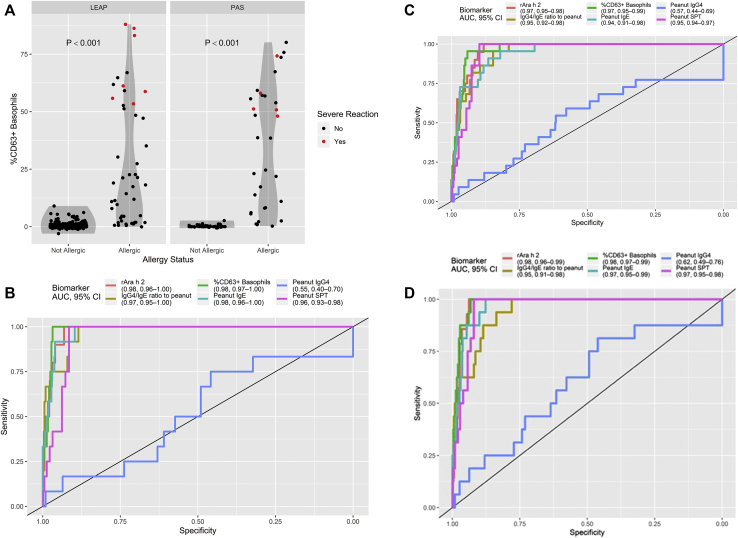
Basophil activation to peanut in participants with PA versus in participants without PA (median = 58.1 [interquartile range = 51.2-74.2] vs median = 10.3 [interquartile range = 1.9-24.2], respectively; *P* < .001) in the PAS and LEAP studies (**A**) and receiver operator characteristic curve for the BAT and other biomarkers to identify LEAP and PAS study subjects at high-risk of developing severe or life-threatening reactions during OFCs, using the Common Terminology Criteria for Adverse Events score (**B**), Ewan and Clark grading (**C**), or medication grading (**D**). *AUC*, Area under the receiver operating characteristic curve.

Participants with severe or life-threatening reactions during OFC had higher proportions of activated basophils in response to peanut than did participants with mild or moderate reactions (Fig 2, *A* and see Table E9 in this article’s Online Repository at www.jacionline.org). Basophil activation in response to anti-FcεRI and anti-IgE (but not fMLP) was higher for severe reactors than for nonsevere reactors (see Fig E4 in this article’s Online Repository at www.jacionline.org). Fig 2, *B-D* illustrate the results for the other biomarkers according to allergic status, highlighting participants with severe or life-threatening reactions.

The BAT showed high accuracy in identifying subjects with severe or life-threatening allergic reactions according to the Common Terminology Criteria for Adverse Events severity score, and it was the single biomarker with the largest area under the receiver operator characteristic curve (0.985 [see Fig 2, *B*]) and therefore with the best discriminatory ability. The optimal cutoff for the BAT had 100% sensitivity and 97% specificity to identify high-risk subjects (Table II). The SPT, Ara h 2–specific IgE level, peanut-specific IgE level, and IgG4/IgE ratio all had 100% sensitivity but lower specificity to predict severity (92%, 93%, 90%, and 88%, respectively). The performance of various tests to identify severe reactors was similar with use of the different systems to classify the severity of allergic reactions (Fig 2, *B* and see Fig E5, *B* and Table E10 in this article’s Online Repository at www.jacionline.org). A multivariable model of reaction severity suggests that after adjustment for the SPT result and level of Ara h 2–specific IgE, the BAT still contributes significantly to the prediction of reaction severity (see Table E11 in this article’s Online Repository at www.jacionline.org).

**Table II:** Optimal cutoffs to classify subjects at high-risk for developing severe allergic reactions and reacting to a low dose (≤0.1 gs) of peanut protein during oral peanut challenge

Parameters	Severe versus nonsevere allergic reactions					Threshold ≤0.1 g vs >0.1 g of peanut protein				
	Cutoff	Sensitivity	Specificity	PPV	NPV	Cutoff	Sensitivity	Specificity	PPV	NPV
BAT (% of CD63^+^ basophils)	48	100 (100-100)	97 (95-98)	41 (32-60)	100 (100-100)	1.7	95 (89-100)	91 (86-96)	43 (35-62)	100 (99-100)
Ara h 2**–**specific IgE (kU/L)	1.4	100 (100-100)	93 (91-98)	22 (18-48)	100 (100-100)	0.1	97 (90-100)	89 (87-95)	38 (34-57)	100 (99-100)
Peanut-specific IgE (kU/L)	5	100 (100-100)	90 (87-98)	18 (15-48)	100 (100-100)	0.4	95 (88 -100)	79 (76-86)	25 (23-34)	100 (99-100)
Peanut-specific IgG4 (μg/L)	175	75 (17-100)	46 (30-99)	3 (2-29)	99 (98-100)	75	82 (67-89)	34 (32-45)	8 (8-10)	96 (94-98)
IgG4/IgE **r**atio	1.6	100 (92-100)	88 (86-98)	16 (14-60)	100 (100-100)	2.1	91 (86-100)	87 (78-92)	35 (25-46)	99 (99-100)
Peanut SPT (mm)	8	100 (100-100)	92 (89-94)	21 (17-27)	100 (100-100)	6	98 (97-100)	95 (91-96)	59 (45-66)	100 (100-100)

Severity was assessed according to the Common Terminology Criteria for Adverse Events scale.^18^ Optimal cutoffs were determined on the basis of the Youden index, which is the distance between the point of inflection of the receiver operator characteristic curve and the reference line (see the Methods section in this article’s Online Repository at www.jacionline.org). Sensitivity, specificity, PPV, and NPV with 95% CIs are indicated for each cutoff.

*NPV*, Negative predictive value; *PPV*, positive predictive value.

### The SPT and BAT were the best predictors of the threshold dose of reactivity to peanut during oral challenges

**Fig 3:**
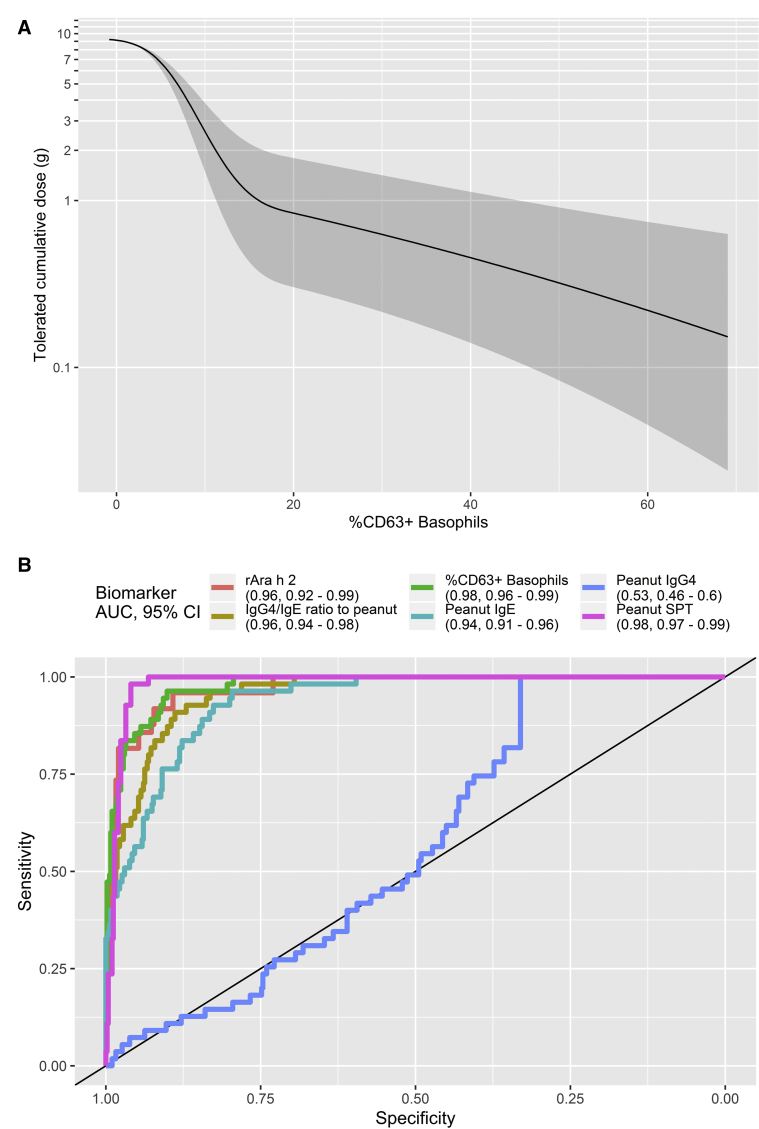
Threshold dose of peanut protein. Extinction curve relating the cumulative peanut threshold dose with the proportion of activated basophils following peanut stimulation (**A**), showing the estimated mean (and 95% confidence band) cumulative amount of peanut tolerated as a function of BAT measurement, and receiver operator characteristic curve for different biomarkers for predicting a threshold less than 0.1 g of peanut protein (**B**). *AUC*, Area under the receiver operating characteristic curve.

**Fig E6:**
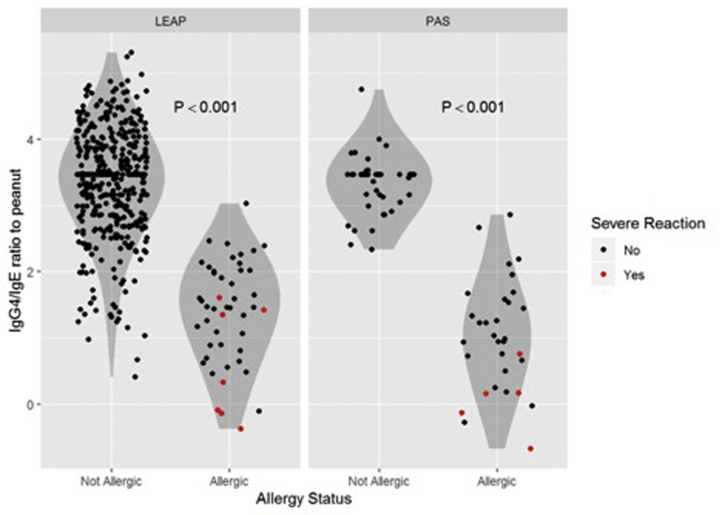


**Fig E7:**
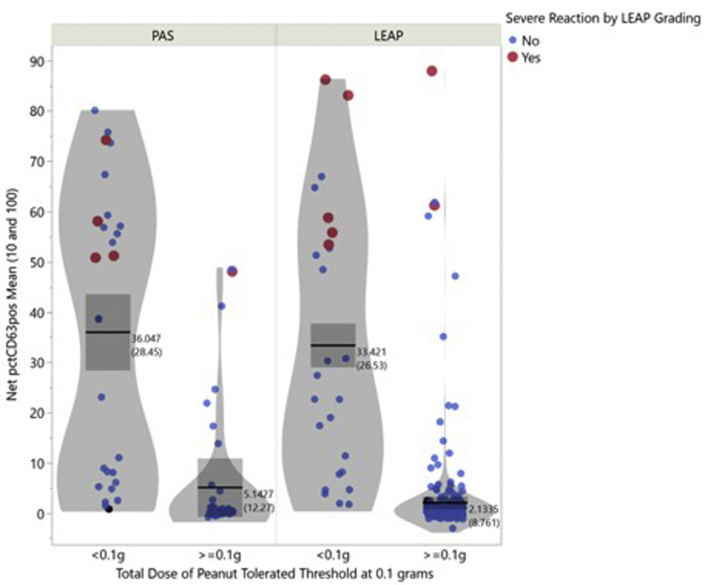


**Fig E8:**
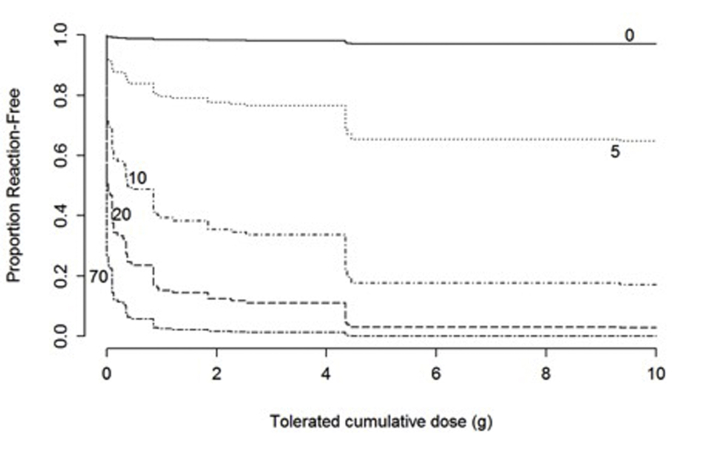


**Fig E9:**
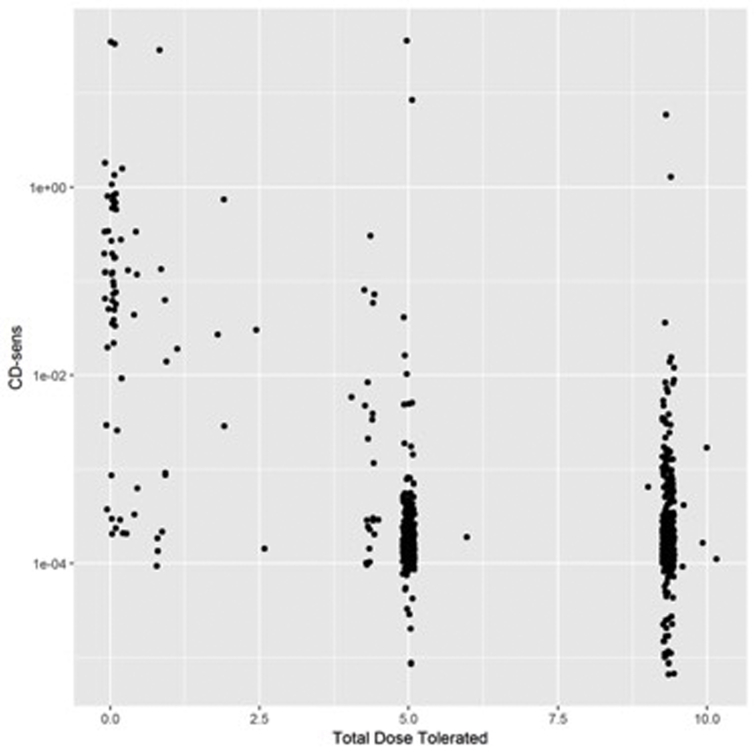


Participants reacting clinically to lower doses of peanut protein had higher proportions of activated basophils (Fig 3 and see Figs E6-E9 in this article’s Online Repository at www.jacionline.org) and higher basophil sensitivity, as measured by CD-sens (see Fig E8). There was no association between lower threshold dose and basophil activation to the positive controls fMLP, anti-IgE, or anti-FcεRI. Fig E7 shows the estimated proportion of participants who remain reaction-free as a function of cumulative peanut dose and reinforces the concept that the higher the proportion of activated basophils, the lower the dose of peanut protein to which patients react during OFC. With use of the receiver operator characteristic curve analyses, the optimal cutoffs for the SPT, level of Ara h 2–specific IgE, and IgG4/IgE ratios also had a very good performance in identifying patients with a cumulative threshold dose of 0.1 g or lower (Table II).

### Models combining different biomarkers to predict severity and threshold dose of allergic reactions during oral peanut challenges

**Fig 4:**
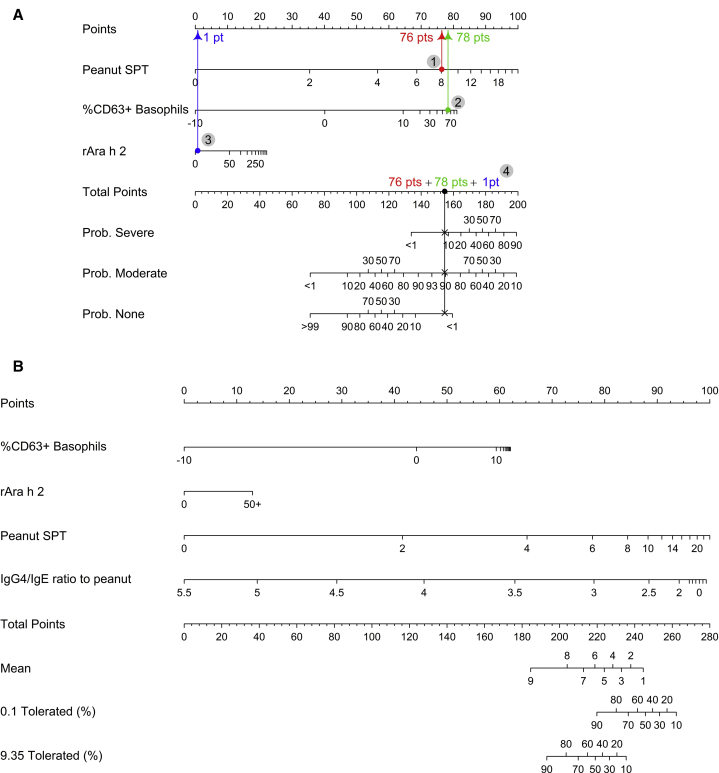
Nomogram for predicting reaction severity using the BAT, the SPT, and level of Ara h 2–specific IgE (**A**) and nomogram for predicting cumulative dose threshold using the BAT, the SPT, level of Ara h 2–specific IgE, and IgG4/IgE ratio **(B)** on the basis of the LEAP and PAS studies when subjects were approximately 5 years of age. Predictions from the models can be made in a clinical setting by simply adding up points earned from each of the variable axes and then using that total to read estimated probabilities from the probability axes. **A,** For example, if we encounter a participant with an rArah h 2 level of of 1.5, a peanut SPT result of 8, and a BAT result of 53, we first find the value 1.5 along the axis associated with rArah h 2 (*second from the top*) and read vertically up to the corresponding point along the top Points axis (*blue arrow*). Similarly, we find the value 8 along the Peanut SPT axis and follow vertically to the Points axis to find that a Peanut SPT result of 8 earns about 76 points (*red arrow*). Similarly, a BAT value of 53 earns about 78 points (*green arrow*). Totaling the points earned from each variable gives 155 points for this participant. We find this total points value on the Total Points axis (*fourth from the bottom*) and imagine a vertical line extending down from that point intersecting each of the probability axes. These points of intersection are the predicted probabilities of falling into each of the severity categories. Given the values for the aforementioned hypothetical participant, we estimate less than a 10% chance of having a severe reaction, a 90% chance of having a moderate reaction, and less than a 10% chance of no reaction. **B,** For example, if we suppose that in addition to the biomarker values seen in (**A**), the participant has a log_10_-IgG4/IgE ratio to peanut of 1.6, the nomogram could be used if we wanted to estimate the mean cumulative tolerated dose, or the probabilities of having mean cumulative doses greater than 0.1 g or 9.35 g given these biomarker values. With a BAT value of 53 we accrue about 62 points, and with an Ara h 2 value of 1.5 we accrue about 5 points; a peanut wheal of 8 gives about 85 points, and an IgG4/IgE ratio of 1.6 gives about 95 points. This individual then has about 247 points in total, which gives an estimated mean cumulative tolerated dose of less than 1 g of peanut, a 45% chance of tolerating more than 0.1 g of peanut, and less than a 10% chance of tolerating more than 9.35 g of peanut. In fact, this individual had a severe reaction during the peanut double-blind placebo-controlled food challenge (DBPCFC) and tolerated 0 g of peanut.

We designed multivariate models combining different parameters to determine the risk of a severe reaction and, with the application of our findings to routine clinical practice in mind, we used these models to generate nomograms (Fig 4A and see Fig E10, *A*-*H* in this article’s Online Repository at www.jacionline.org). We performed internal validation of these models by using the bootstrap to correct for the bias of using the same data to fit and validate the model. Although the performance of both multivariate models was good (Table III), on the basis of calibration, the model including the BAT more accurately predicted the probability of severe or life-threatening reactions than did either the model without the BAT or the individual tests.

**Table III:** Model performance measures for reaction severity

Model	C-Index	Absolute calibration error			
		Moderate or higher		Severe/life-threatening	
		Mean	90th percentile	Mean	90th percentile
Multivariable (BAT)	0.991	0.5	0.9	0.3	0.3
Multivariable (no BAT)	0.985	0.3	1	0.5	0.3
SPT	0.985	0.5	1.6	0.5	0.6
Ara h 2–specific IgE	0.951	4.1	7.3	0.3	0.1
BAT	0.951	1.6	4	0.3	0.2
IgE to peanut	0.914	4.4	14.3	0.6	1.7
IgG4 to peanut	0.563	1.1	1.7	0.4	1

The C-index measures the model’s rank discrimination (ie, the ability of the model to correctly rank subjects), with 1 being perfect discrimination and 0.5 indicating a coin toss model. The calibration error measures how far apart in percentage points model-based predictions are from true predictions, as in a calibration curve, for each ordinal model cut point. The mean absolute calibration error is the absolute prediction error averaged across all predictions made, with an analogous definition for the 90th percentile of absolute prediction error. In each case, the smaller the error, the better.

**Fig E11:**
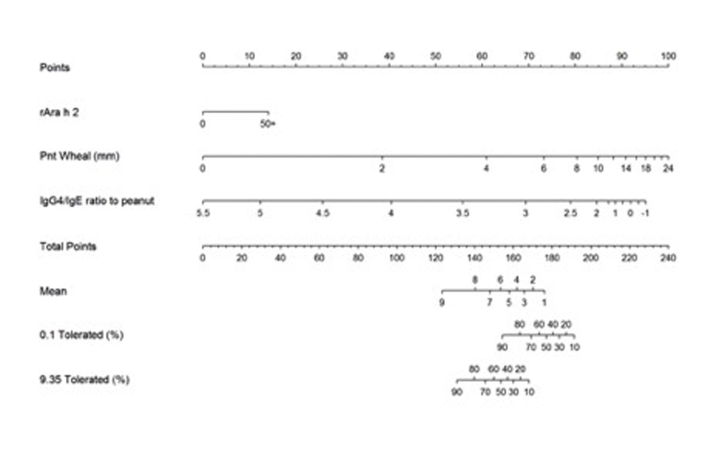


A multivariable model for threshold built using the best predictors for cumulative threshold dose and including the BAT (the SPT, the BAT, level of Ara h 2–specific IgE, level of peanut-specific IgE, and IgG4/IgE ratio) was superior to the model without BAT and outperformed models based on any of the predictors individually (Table IV). The nomogram in Fig 4, *B* depicts this multivariable model and can be used to make predictions of threshold of future reactions during PA testing. A similar nomogram without BAT is shown in Fig E11 (in this article’s Online Repository at www.jacionline.org).

**Table IV:** Model performance measures for cumulative dose threshold

Model	C-Index	Absolute calibration error					
		0.1 g		5.0 g		9.35 g	
		Mean	90th percentile	Mean	90th percentile	Mean	90th percentile
Multivariable	0.981	1.6	5.6	0.8	2.7	0.3	0.2
Multivariable (no BAT)	0.980	2.2	5.1	1	3	0.7	1.2
SPT	0.972	1.4	8.9	0.8	3.2	0.6	2
BAT	0.938	1.8	7.8	1	2	0.9	2.8
Ara h 2–specific IgE	0.938	3.6	13.4	6.2	9.6	6.9	12.6
IgG4/IgE ratio to peanut (log 10)	0.932	3.1	8	3.6	6.4	2.1	2.8
IgE to peanut	0.899	4	10.1	6	16.8	6.6	20.7
IgG4 to peanut	0.565	1.7	2.1	2.6	4.8	3	4.2

The C-index measures the model’s rank discrimination (ie, the ability of the model to correctly rank subjects), with 1 being perfect discrimination and 0.5 indicating a coin toss model. The calibration error measures how far apart in percentage points model-based predictions are from true predictions, as in a calibration curve, for each ordinal model cut point. The mean absolute calibration error is the absolute prediction error averaged across all predictions made, with an analogous definition for the 90th percentile of absolute prediction error. In each case, the smaller the error, the better.

**Fig E12:**
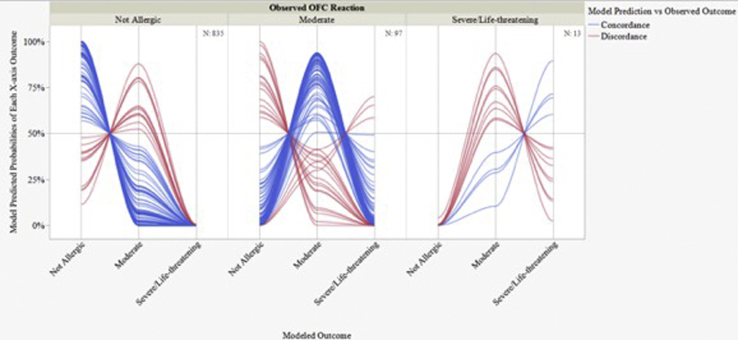


Internal validation reflects mostly the performance of the model within a population similar to the population of the present study, and external validation is important to assess the model in more general settings. External validation of the severity and the threshold models was performed by using an independent cohort recruited from 2 specialized pediatric allergy outpatient clinics in London (see Table E2). Although this data set was limited by not including severe cases, the model did accurately predict those without allergy and mild or moderate reactions. For more details, see the Results section in the Online Repository, Table E12 and Figs E12-E14 in this article’s Online Repository at www.jacionline.org.

**Fig 5:**
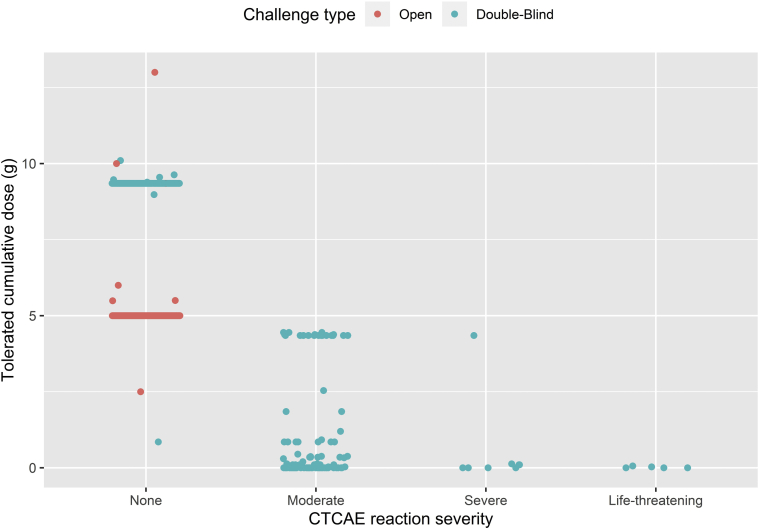
Relationship between severity and threshold of allergic reactions to peanut. Points represent individual subjects submitted to either double-blind placebo-controlled (*blue points*) or open (*red points*) peanut challenges in the LEAP studies. The Spearman rank correlation between cumulative tolerated dose and severity of Common Terminology Criteria for Adverse Events (CTCAE)-graded reactions is –0.96 (*P* < .001) at 60 months and –0.94 (*P* < .001) at 72 months. When these correlations were computed, open challenges not ending in a reaction were given an imputed tolerated cumulative dose of 9.35 g.

We observed a relationship between clinical severity and cumulative threshold dose in patients with PA: the lower the cumulative dose of peanut protein tolerated, the greater the severity of allergic reactions to peanut during incremental peanut challenge (Fig 5). A test of the association between the risk of having a reaction during OFC and percentage of CD63^+^ basophils was found to be significant (*P* < .001).

## Discussion

The OFC is the criterion standard to diagnose PA, to confirm eligibility for clinical trials, and to assess the response to treatments of patients who are known to be allergic. Although OFC is generally safe, the severity of allergic reactions is unpredictable and allergic symptoms can cause significant discomfort and anxiety to patients and families.^1^^,^^2^ Furthermore, OFCs are costly and time-consuming, and they require a lengthy visit that may be obviated by objective, safer, cheaper, and more convenient biomarkers. Herein, we tested a large number of very well-characterized subjects who participated in the LEAP study and associated studies, and we confirmed that the BAT is an excellent biomarker of PA and that the BAT and other biomarkers are able to identify patients at risk of reacting to small amounts of peanut protein and developing severe symptoms during peanut OFC. The multivariate model that combines various biomarkers can be computed by using nomograms to identify individuals at high-risk of a severe or life-threatening reaction or with a low threshold of reactivity during OFC in clinical practice. There are very few data in the literature combining different biomarkers to determine diagnostic accuracy, and we have demonstrated in this article that a multivariate model predicts severity and threshold. We have also demonstrated an inverse correlation between threshold and severity of allergic reactions to peanut.

We have shown, for the first time, that the SPT, specific IgE level, level of Ara h 2–specific IgE, and BAT are predictors of severity of allergic reactions during OFC. The optimal cutoffs for these tests showed 100% sensitivity to identify severe reactors, meaning that all subjects with severe reactions had results for these tests that were above the defined cutoff. Furthermore, this is the largest cohort tested with the BAT to peanut by using a method that was previously validated.^11^ Study participants were very well characterized with a rigorous methodology.^15^^,^^16^ The BAT proved to be an accurate diagnostic test that is able to confirm PA with high specificity, which is in agreement with our previous findings.^11^ Being able to identify patients with PA who are at risk of reacting to a low dose of the allergen and/or at risk of developing life-threatening reactions can improve current management of patients with PA. Previous studies, including our own peanut study using the exact same BAT methodology,^9^ have shown a direct correlation between the severity of symptoms and basophil activation and an inverse correlation between the threshold of reactivity and basophil activation.^9^^,^^12^^,^^13^ More recently, another study of patients undergoing OFC to peanut identified the BAT as the best biomarker for severity of allergic symptoms^22^ when using the same BAT parameter that we had previously chosen as the preferred outcome for the BAT.^9^ In the present study, we also assessed basophil sensitivity as expressed by CD-sens, which previously was the marker that best reflected low threshold dose, and we found that basophils of patients reacting at lower doses of peanut protein in the OFC reacted to lower concentrations of peanut extract *in vitro*, thus displaying a higher CD-sens. Of note, the associations between SPT and threshold and between SPT and severity may be biased toward a stronger association, given that the clinician knows the results of the SPT before doing the challenge. Conversely, because the clinician is not aware of the BAT results, the BAT may have less of an intrinsic bias and may constitute a more robust biomarker of severity and threshold compared with, for instance, the SPT result, which was known to the clinical team performing the OFC.

The observation that a higher proportion of activated basophils is associated with more severe reactions and a lower threshold raises the question as to whether severity is linked to threshold. Although clinically this association is difficult to establish owing to the ethical constraints of continuing OFC after an allergic reaction has started, we have previously shown that *in vitro* BAT markers of severity and threshold are strongly correlated. This strong correlation is probably due to the ability to stimulate basophils *in vitro* with higher concentrations of allergen than are possible *in vivo* during OFC for ethical reasons. In the LEAP study protocol, OFC was started at a relatively high dose of peanut protein (0.1 g in most patients and 0.033 g in high-risk patients^15^^,^^23^) because this was a diagnostic OFC, whereas most OFCs start with less than 10 mg of peanut protein and, according to other threshold studies, a dose of 100 mg approaches the eliciting dose for 30% to 50% of the population with PA; thus, the LEAP study is poorly designed to look at threshold. However, because OFC started at a relatively high dose, we were able to see a strong correlation between threshold and severity that would otherwise not have been possible, as the OFC would have been stopped at the first signs of an allergic reaction at lower doses. The fact that BAT in this study is related to both severity and threshold further indirectly supports a link between low threshold and increasing severity of allergic reactions.

Having a high proportion of activated basophils in response to the allergen does not necessarily mean that the patient will have a severe allergic reaction when accidentally exposed to the allergen, but it allows identification of high-risk patients who deserve a closer follow-up. This is in line with the findings of other studies and the notion that severity of allergic reactions depends on a multitude of factors,^24^ of which the effector cell biology is only one. The fact that the basophil response to IgE-mediated positive controls (but not to the non–IgE-mediated positive control fMLP) was higher in severe reactors than in patients with mild or moderate reactions may suggest that differences intrinsic to the IgE-mediated pathway in basophil effector cells can influence the severity of symptoms. The same was not observed for threshold dose or for allergic status to peanut.

Previous studies investigating the SPT and testing of specific IgE as biomarkers for severity showed conflicting results—some with positive findings^5^^,^^6^^,^^9^^,^^25^, ^26^, ^27^, ^28^, ^29^, ^30^ and others with negative findings,^7^^,^^8^^,^^31^, ^32^, ^33^, ^34^, ^35^ possibly because of differences in study design, patients included, and OFC protocols. In contrast, in our study, tests other than the BAT, such as the SPT, Ara h 2–specific IgE, peanut-specific IgE, and IgG4/IgE ratios, performed well in identifying severe reactors. For instance, an SPT result of 8 mm or larger can be useful clinically to identify patients at greater risk. The SPT was in fact the best biomarker for threshold followed by BAT. However, better than any individual test were the multivariate models, as shown by the higher C-value. We would like to underscore that statistically speaking, improving on an already high C-index is “more significant” than the same level of improvement of a lower C-index. Multivariate models take into account the correlation between metrics, and this adds robustness to the predictions of individual tests. We included the results of tests in a multivariate model to generate user-friendly nomograms to predict the probability of a serious adverse event during peanut OFC for a given patient. This may be different from allergic reactions in the community, where additional factors that are controlled for in the context of OFC can contribute to the outcome (eg, uncontrolled asthma, concomitant infection, and other cofactors)^5^^,^^36^ The nomograms can be very useful clinically, with the ability to identify high-risk patients who may benefit from a different OFC protocol, starting with lower doses with more careful clinical monitoring (eg, cannulation before the start of OFC, higher clinician-to-patient ratio during OFC) or determining when OFC should be dispensed with. Instead, confirmation of PA could be done with an *in vitro* test such as the BAT or with a combination of tests using the nomogram. Because of the limitations of the external validation cohort used in the present study, further rigorous external validation is needed before applying these results more broadly, namely, with other food allergens and in other patient populations.

A proportion of participants could not be tested with the BAT for logistic reasons, namely, failure to collect blood, appointments too late in the day, or too many appointments per day to allow for performance of the BAT in a timely fashion. This limitation highlights the need to simplify the BAT procedure and have an organized system to overcome these barriers in clinical practice, should the BAT be used as a biomarker in a real-life scenario. The differences observed between patients who were and were not tested with the BAT reflect the fact that tested patients were more likely to be allergic to peanut. As patients without allergy do not ever have a positive response to allergen on the BAT, we believe that this potential bias has not affected the results but rather has granted the ability to capture a high-risk population.

In all, 14% percent of samples were not considered in the analyses because of nonresponder basophils, which is a known limitation of BAT. However, nonresponders would not lead to misdiagnosis of high-risk patients, as these patients required an OFC to confirm or exclude PA, and thus, we would not exclude allergy on the basis of nonresponder status. In clinical practice, there are patients for whom we do not have results for the other tests. For instance, a significant proportion of children do not react on the SPT, when during the pollen season, they cannot stop taking antihistamines, and in such situations, the BAT is particularly useful because it is not affected by antihistamines.^37^ The nonresponder status was not consistent over time, with some participants having nonresponder basophils at 1 time point (LEAP/LEAP-On studies) and not the other, which is in line with experimental data showing that the nonresponder status results from transient changes in cell signaling proteins and can be reversed in different culturing conditions, namely, in the presence of IL-3.^38^^,^^39^ The model without the BAT could be used to identify patients at risk of severe reactions and low threshold, in the case of nonresponders.

In summary, by using a large cohort of well-characterized patients, we confirmed that the BAT is a biomarker of PA and of the severity and threshold of allergic reactions to peanut during OFC. All of the other markers had a very good sensitivity but lower specificity to predict severity, whereas the SPT was a better biomarker for threshold. The best predictive approach both for severity and for threshold was to combine tests in a multivariate model. By using novel models that integrate various biomarkers, we are able to generate nomograms that could be used in clinical practice to identify patients with allergy who are at higher risk of experiencing severe allergic reactions or of reacting to a low dose of peanut protein and offer them a more personalized management plan and follow-up.

**Key messages**

• The BAT was useful to diagnose PA and to predict severity and threshold of reactivity to peanut in the LEAP studies.
• The SPT and level of Ara h 2–specific IgE were also useful biomarkers of severity and threshold in the LEAP cohorts.
• Multivariate models combining basophil activation with other tests using a nomogram identified children with severe allergic reactions and low threshold of reactivity during oral peanut challenges.

## Figures and Tables

**Fig E5A:**
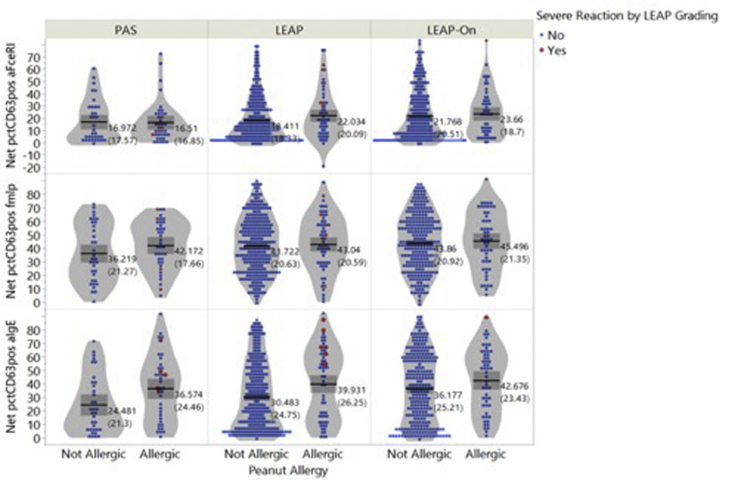


**Fig E5B:**
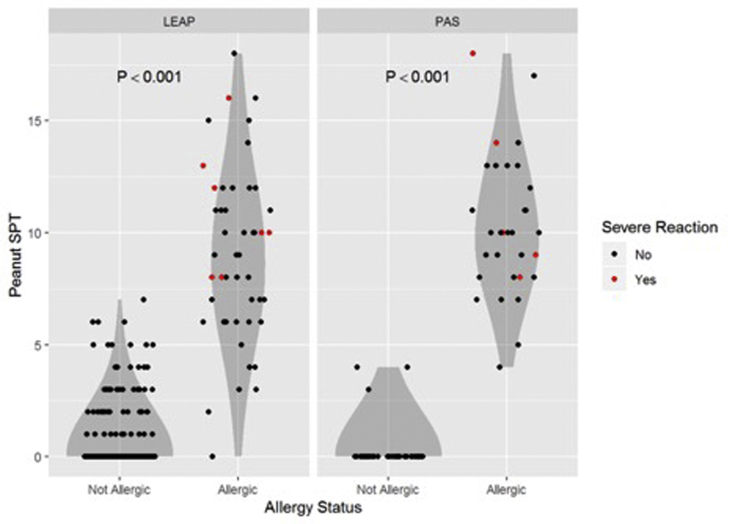


**Fig E5C:**
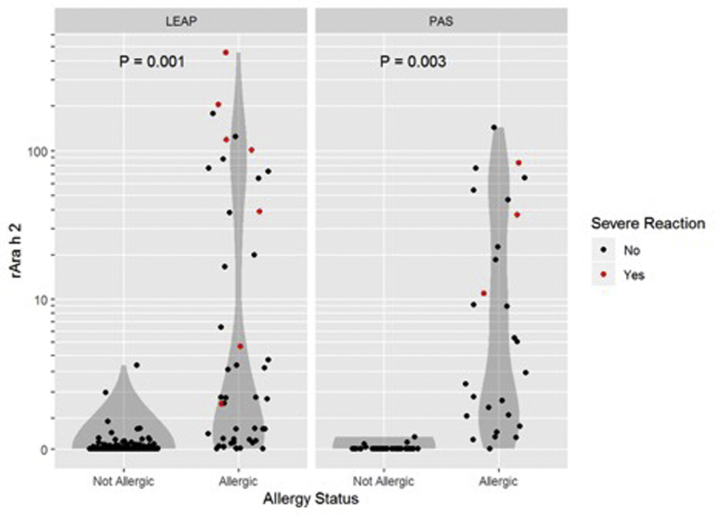


**Fig E5D:**
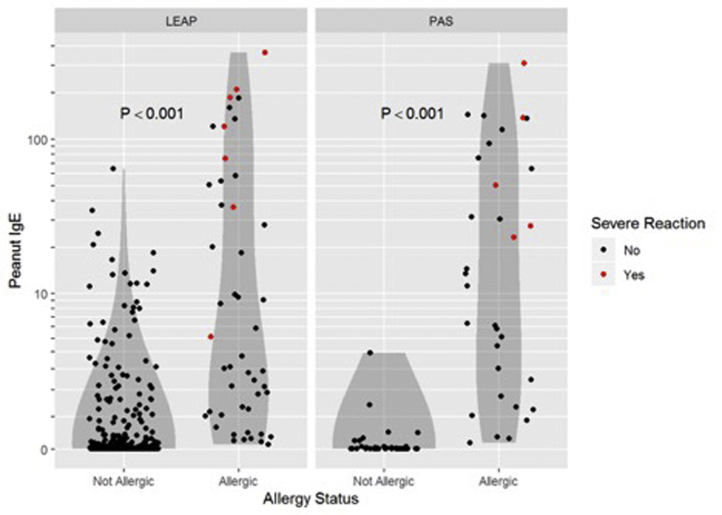


**Fig E5E:**
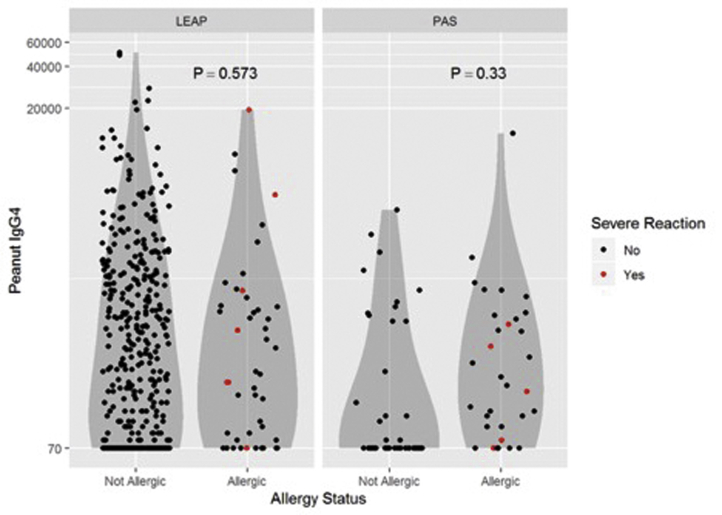


**Fig E10A:**
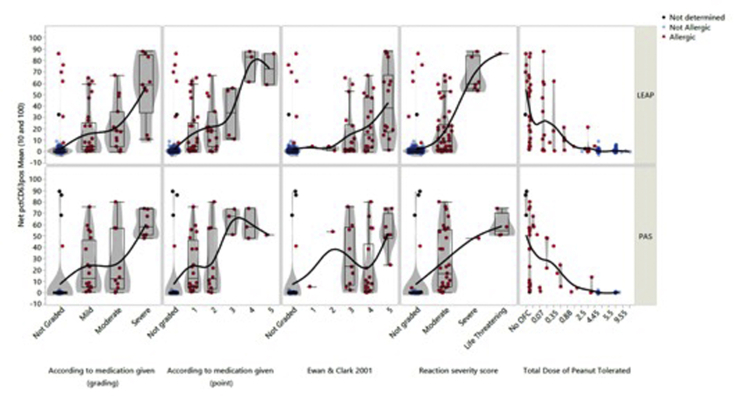


**Fig E10B:**
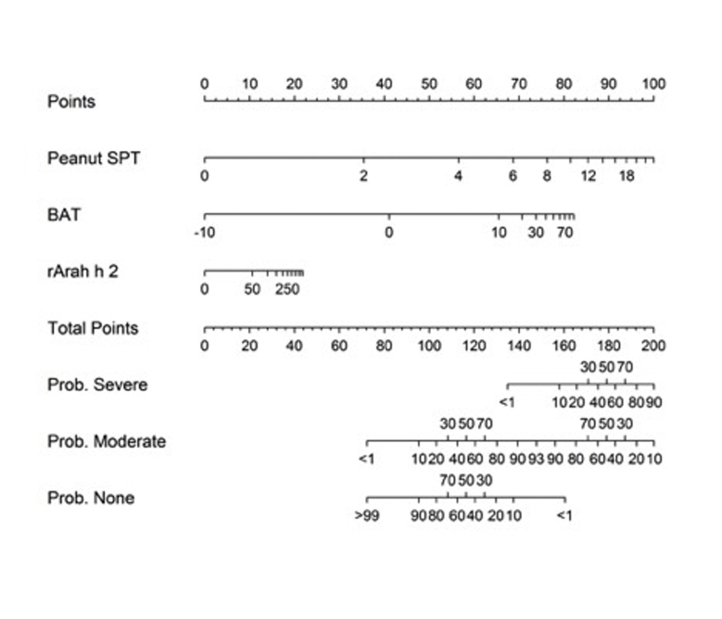


**Fig E10C:**
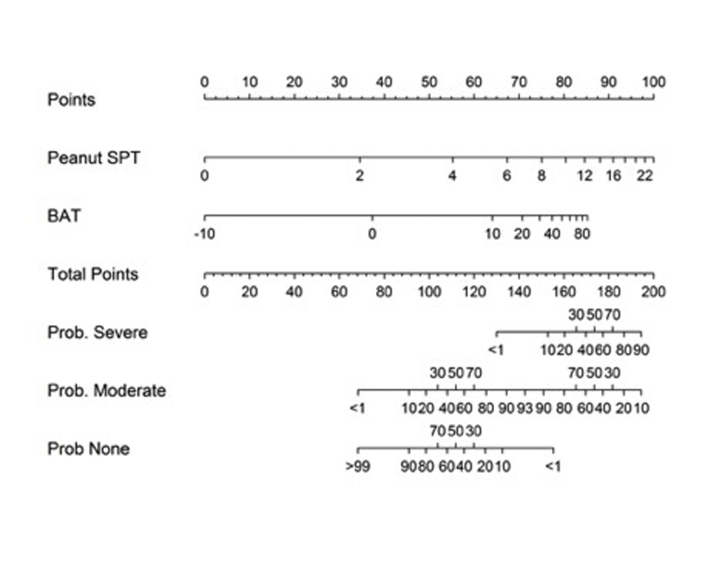


**Fig E10D:**
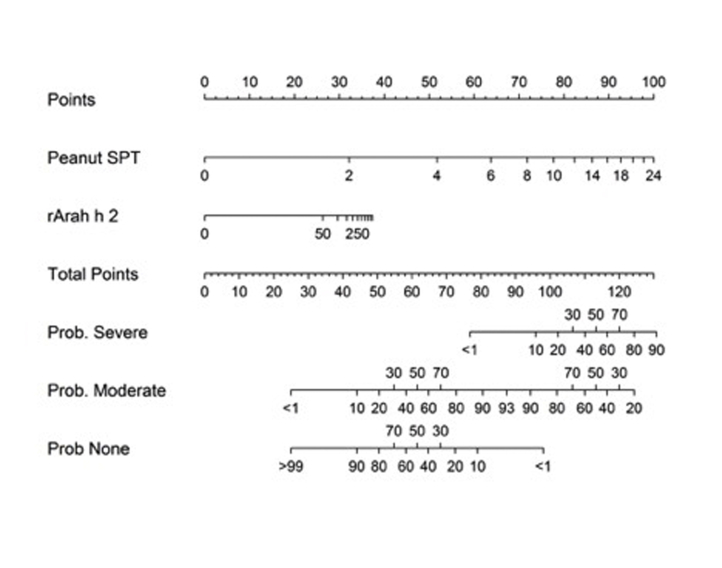


**Fig E10E:**
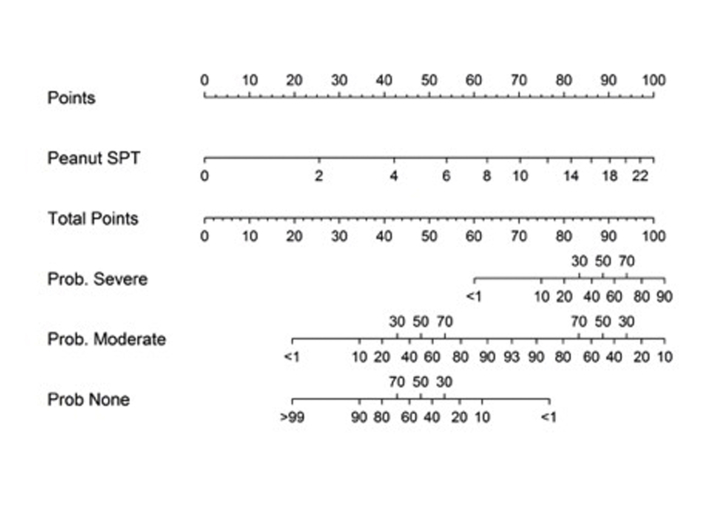


**Fig E10F:**
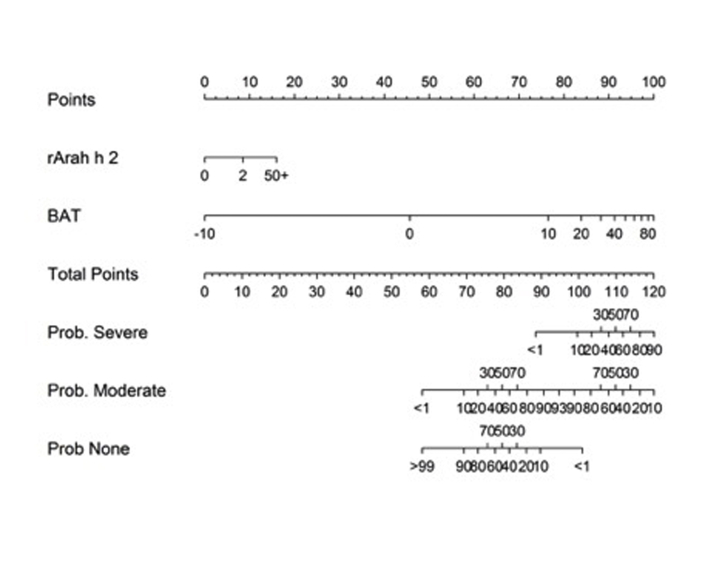


**Fig E10G:**
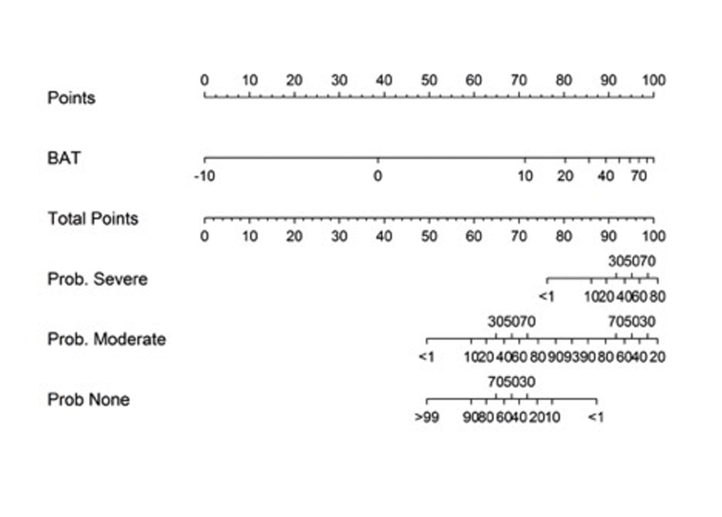


**Fig E10G:**
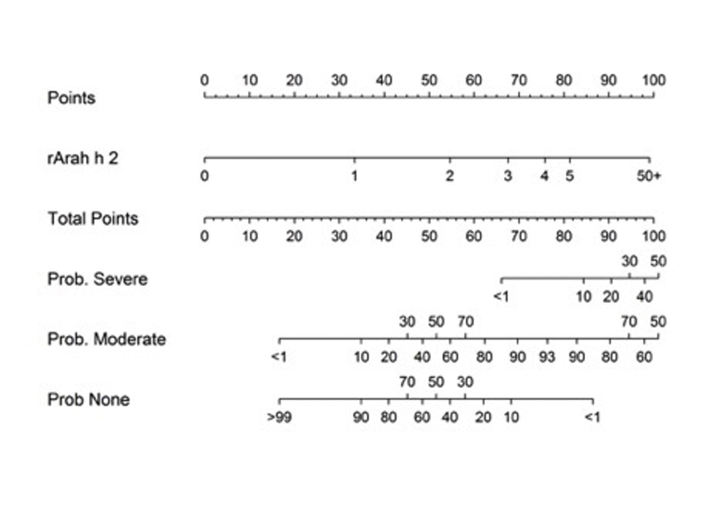


**Fig E10H:**
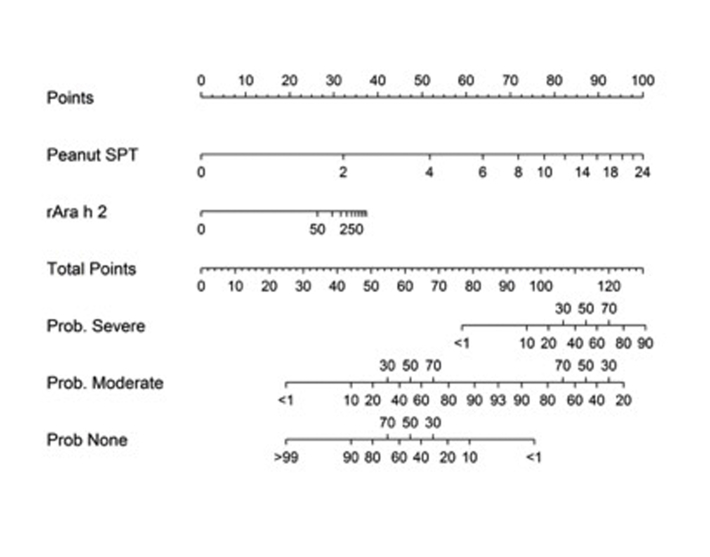


**Fig E13A:**
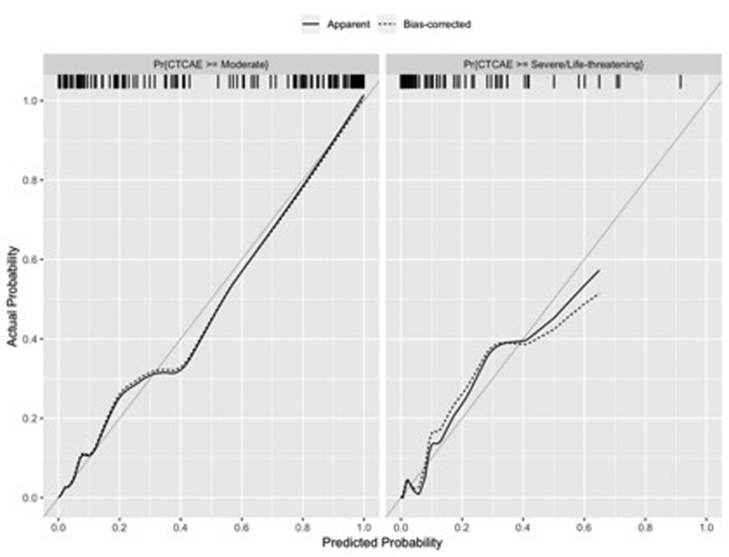


**Fig E13B:**
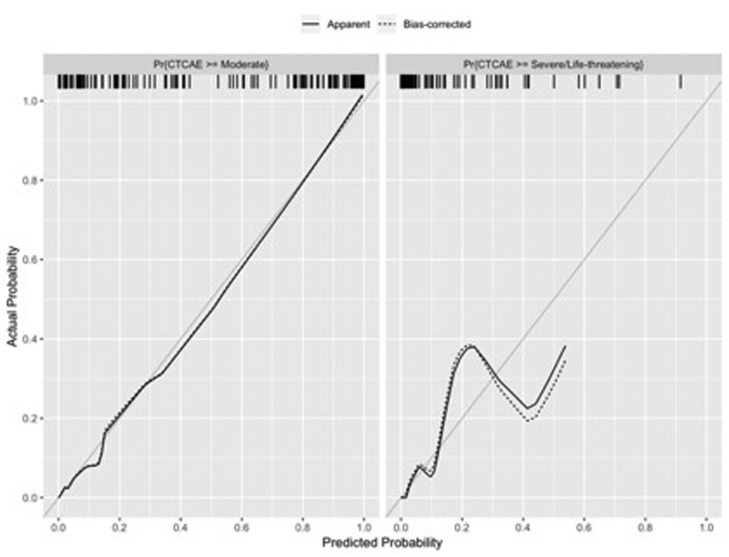


**Fig E14A:**
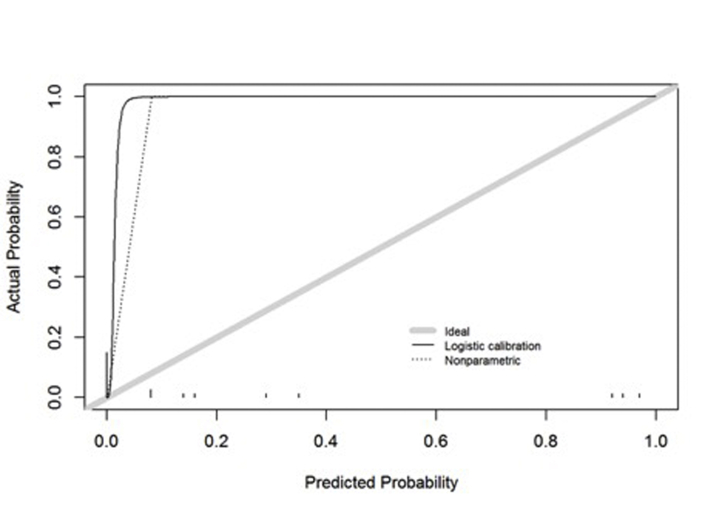


**Fig E14B:**
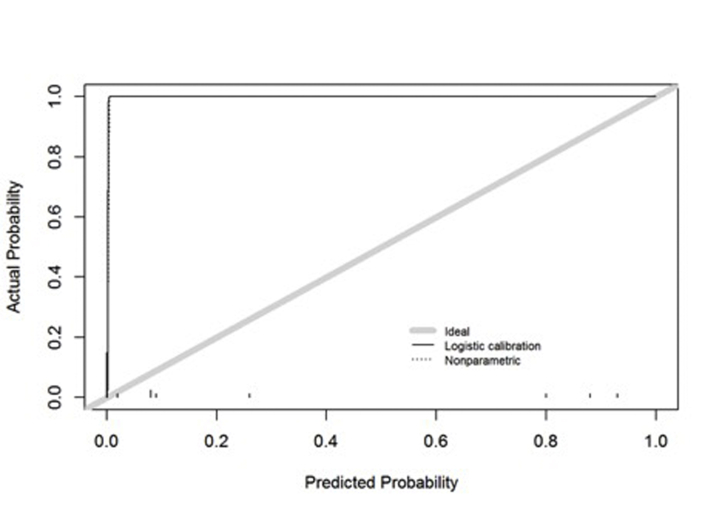


## Supplementary data

Online Repository
