# Supplementary material for: Biomarkers of severity and threshold of allergic reactions during oral peanut challenges
Source: J Allergy Clin Immunol. 2020 Aug;146(2):344–55. doi: 10.1016/j.jaci.2020.03.035 (PMC7417812; doi:10.1016/j.jaci.2020.03.035)
Supplement: Online Repository [file mmc1.docx]

**Online Repository Material:**

**Biomarkers of severity and threshold of allergic reactions during oral peanut challenges**

Alexandra F. Santos, MD PhD^1,2,3,4^; George Du Toit, MD FRCPCH^1,2,3*^; Colin O’Rourke, MS^5*^; Natalia Becares, PhD^1,2,4^; Natália Couto-Francisco, MSc^1,2,4^; Suzana Radulovic, MD^1,2,3^; Ekaterina Khaleva, MD^3^; Monica Basting, MA^1,2^; Kristina M. Harris, PhD^6^; David Larson, PhD^6^; Peter Sayre, MD PhD^7^; Marshall Plaut, MD^8^; Graham Roberts, DM FRCPCH^9^; Henry T Bahnson, MPH^5^; Gideon Lack, MD FRCPCH^1,2,3,4^.

**These authors contributed equally to the manuscript.*

^1^Department of Women and Children’s Health (Pediatric Allergy), School of Life Course Sciences, Faculty of Life Sciences and Medicine, King’s College London, London, UK

^2^Peter Gorer Department of Immunobiology, School of Immunology and Microbial Sciences, King’s College London, London, UK

^3^Children’s Allergy Service, Guy’s and St Thomas’ Hospital, London, UK

^4^Asthma UK Centre in Allergic Mechanisms of Asthma, London, UK

^5^Immune Tolerance Network, Benaroya Research Institute, Seattle, USA

^6^Immune Tolerance Network, Bethesda, MD, USA

^7^Division of Hematology–Oncology, Department of Medicine, University of California, San Francisco, San Francisco, USA

^8^National Institute of Allergy and Infectious Diseases, Bethesda, USA

^9^David Hide Asthma and Allergy Research Centre, St Mary’s Hospital, Isle of Wight; NIHR Biomedical Research Centre, University Hospital Southampton NHS Foundation Trust and Clinical and Experimental Sciences Academic Unit, University of Southampton Faculty of Medicine, Southampton, United Kingdom

**Corresponding authors:**

Name: Alexandra F. Santos

Postal address: Department of Pediatric Allergy, 2nd floor, South Wing, St Thomas’ Hospital, Westminster Bridge Road, SE1 7EH London, United Kingdom

Telephone: +44 (0) 20 7188 6424

Fax number: +44 (0) 20 7403 8640

Email address: alexandra.santos@kcl.ac.uk

Name: Gideon Lack

Postal address: Department of Pediatric Allergy, 2nd floor, South Wing, St Thomas’ Hospital, Westminster Bridge Road, SE1 7EH London, United Kingdom

Telephone: +44 (0)2071889730

Fax: +44 (0)2071889782

Email address: gideon.lack@kcl.ac.uk

## E-methods:

**Statistical analyses**

Youden’s J statistic was used to derive a cut point for classifying severe reactions based on the Ewan, medication grading severity scale and CTCAE reactions severe grading methods. The operating characteristics of “optimal” cut points were described and 95% confidence intervals were computed using the bootstrap with 2,000 resamples. Prior to modeling, the predictors were singly imputed using the methods implemented in the R^1^ package Hmisc^2^. Imputation was based on Ara h 2, anti-IgE, SPT, IgG4, aFceRI, anti-IgE, fMLP, and cumulative dose threshold. In order to achieve better predictive performance, model assumptions of linearity were relaxed by including continuous predictors as flexible restricted cubic spline terms. Both internal and external validation were performed. Internal validation was done using bootstrap bias-corrected model performance measures to correct for optimism due to both fitting and assessing performance using the same data.

CD-sens is defined as the reciprocal of the peanut protein concentration value at which half of the maximal BAT level is achieved. BAT level as a function of log_10_-peanut concentration was modeled for each participant using a logistic growth function. This type of function as the general form shown in Figure E1 with shape controlled by three parameters ­ maximum value (**A**), the midpoint (**M**) at which the function has achieved half its maximum value, and a value (**S**) controlling the steepness of the curve. From each participant’s estimated BAT growth, CD-sens was then calculated as 10^-M^.

While the observations associated with the LEAP-On study were excluded when performing ROC analyses, since all LEAP-On subjects were originally LEAP subjects, they were included for the purposes of building multivariable prediction models. Despite not being independent samples it is often advantageous to use all available data when forming a prediction model, which was corroborated through comparisons with models refit using only observations from the LEAP and PAS studies. There are differences between the statistics reported from the ROC analyses of biomarkers to predict severe vs. non-severe reactions and the C-statistics from the multivariable models. The C-statistics produced by the ROC analyses and the model relate to fundamentally different outcomes. Specifically, the ROC analyses predict a dichotomous outcome (severe vs. not severe) whereas the models predicts an ordinal (no reaction, mild/moderate, severe/life-threatening). In addition, a C-statistic generalizes the idea of the AUC_ROC_ to situations when the outcome is not binary (e.g. ordinal). When binary, the C-statistic and AUC_ROC_ are the same, but in cases where the outcome is not binary the C-statistics does not relate to any standard AUC_ROC_. The models include the ability for biomarkers to act nonlinearly (and possibly non-monotonically) with an outcome.

**E-Results**

## Validation of predictive models as biomarkers of severity and as biomarkers of threshold

The multivariable model with BAT had a high concordance index of 0.99 for predicting CTCAE reaction severity grade. Predicted allergy categories based on this model displayed a high level of concordance, with 96% (906/945) of the predicted severity levels being concordant with their observed severity levels (Figure E12). Figure E13A shows a calibration plot depicting the accuracy of the model in estimating the probability of a moderate or more severe reaction and the probability of a severe/life-threatening reaction. A perfectly calibrated model will show a calibration curve that follows the diagonal line. In our data we see for the probability of having a moderate or more severe reaction an average absolute prediction error of about 0.5%, and prediction errors greater than 1.3% happened about 10% of the time. For the probability of having a severe or life-threatening reaction the average prediction error was about 0.3% and prediction errors greater than 0.1% happened about 10% of the time.

The multivariate model without BAT had a concordance index of 0.99. Figure E13B shows the calibration curves for this model. The mean absolute prediction error for the probability of having a moderate or more severe reaction is 0.2% and prediction errors greater than 1% happened about 10% of the time. When predicting the probability of having a severe or life-threatening reaction we saw an average prediction error of about 0.4%, with prediction errors larger than 0.4% happening about 10% of the time.

External validation of the model of reaction severity was done using data from patients in a clinical setting (Table E2). This clinical cohort was comparable to the LEAP participants in age and the fact that were mostly atopic children being assessed for peanut allergy but differed in the fact that, because they were challenged for clinical reasons in the cohort recruited from the outpatients’ clinic, the proportion of positive challenges and severe reactions was lower than in the LEAP, LEAP-On and PAS studies in which children were challenged irrespective of the peanut sensitization status and previous history of reacting to peanut. Due to low rates of severe reactions, none were observed within the validation cohort. This makes a thorough validation of the model of reaction severity difficult, but we can get some sense of model performance from the 9 moderate graded reactions. Figure E14 show calibration curves for the multivariable models with and without BAT. Both models assign participants with no reaction a low risk score and participants with moderate reactions a high-risk score. This leads to perfect concordance, where all reactive participants are ranked higher than non-reactive participants, but to calibration plots that depict what appears to be very poor performance with large errors.

Modeling of the reaction severity outcome was done assuming ordinal reaction severity categories, i.e. no reaction < Moderate < Severe/Life-threatening. Only ‘no reactions’ and ‘moderate reactions’ outcomes were seen in the validation cohort, and we acknowledge this limitation. However, we believe the validation set is still important since there is both an absence of subjects with severe reactions and an absence of subjects with very high BAT values. Moreover, the validation model shows concordance among those who did not react or had moderate reactions. Therefore, while the dataset would be better if it contained severe reactions, it provides valuable information in the context of external validation.

External validation of the threshold model was done using data from the same patients recruited from clinic (Table E2). Results are shown in Table E11.

**E-Tables**

**Table E1.** Study cohorts included in the study. Infants screened for entry into the LEAP study who were not included were invited to participate in the PAS study, in which peanut allergy was assessed at approximately 5 years of age, following the same procedures as for the LEAP Study. Group I did not satisfy the inclusion criteria of the LEAP Study (i.e. did not have severe eczema or egg allergy); Group IV was considered to be peanut allergic given a SPT to peanut ≥5 mm; Group V, was excluded for other reasons. The LEAP-On Study was the follow-on study of the LEAP study, in which participants from both arms of the study (consumers and avoiders) avoided peanut for one year.

| LEAP Screening Study^1^ | Definition | Assessment at  approx. 5Y of age | Assessment at  approx. 6Y of age |
| --- | --- | --- | --- |
| Group I | Eczema not severe | PAS study | - |
| Group II | Severe eczema or egg allergy  SPT-negative | LEAP Study^2^ | LEAP-On Study^3^ |
| Group III | Severe eczema or egg allergy  SPT-positive |  |  |
| Group IV | SPT ≥5 mm | PAS study | - |
| Group V | miscellaneous |  |  |

## Table E2. Demographic and clinical characteristics of the external validation cohort recruited from two specialized Pediatric Allergy clinics in London, UK.

|  | | **Peanut allergic**  **(N = 29)** | **Peanut-sensitized**  **non-allergic**  **(N = 48)** | **Non-peanut-sensitized**  **non-allergic**  **(N = 11)** |
| --- | --- | --- | --- | --- |
| Male Gender | | 23 (79.3%) | 30 (62.5%) | 7 (63.6%) |
| Atopic eczema^2^ | | 15 (51.7%) | 26 (54.2%) | 7 (63.6%) |
| Results of OFC to peanut | Not Done | 20 (69.0%) | 7 (14.6%) | 10 (90.9%) |
|  | Negative | 0 (0.0%) | 41 (85.4%) | 1 (9.1%) |
|  | Positive | 9 (31.0%) | 0 (0.0%) | 0 (0.0%) |
| Participants with severe or life-threatening reactions during OFC  (% of positive OFC) | | 0/9 (0.0%) | 0/0 | 0/0 |
| Threshold dose of reaction during OFC | | 2.7 (3.6, N=9) | (N=0) | (N=0) |
| SPT (mm) | | 8.7 (4.5) | 2.9 (2.6) | 0.0 (0.0) |
| P-sIgE (KU/L) | | 101.39 (149.09) | 4.74 (8.08) | 0.02 (0.02) |
| Ara h 1-sIgE (KU/L) | | 33.16 (53.57) | 0.70 (2.34, N=47) | 0.01 (0.01) |
| Ara h 2-sIgE (KU/L) | | 44.81 (64.64) | 0.16 (0.28, N=47) | 0.02 (0.02) |
| Ara h 3-sIgE (KU/L) | | 12.42 (24.46) | 0.34 (1.08, N=47) | 0.01 (0.01) |
| Ara h 8-sIgE (KU/L) | | 3.28 (10.88) | 4.14 (11.35, N=46) | 0.01 (0.01) |
| Ara h 9-sIgE (KU/L) | | 0.44 (1.24) | 0.92 (2.62, N=47) | 0.01 (0.0) |
| P-sIgG4 (KU/L) | | 497.2 (627.3) | 662.1 (1029.5, N=47) | 74.5 (112.2) |
| IgG4/IgE Ratio (log_10_) | | 36.6 (82.8) | 147.2 (382.2, N=47) | 1660.4 (2396.1) |
| BAT to peanut  (% CD63+ basophils) | | 34.5 (26.1) | 1.6 (4.0) | 0.7 (1.3) |
| BAT to anti-IgE  (% CD63+ basophils) | | 42.3 (23.7) | 35.3 (22.3) | 26.5 (28.0) |
| BAT to fMLP  (% CD63+ basophils) | | 44.6 (23.3) | 39.3 (18.0) | 40.5 (19.3) |

Note: Summaries are n/N (%) or Mean (SD)

## Table E3. Doses of peanut protein in the incremental double-blind placebo-controlled oral food challenge protocol. Placebo doses were randomly interspersed. Open challenges were done using a cumulative dose of 5g of peanut protein *Additional starting dose for high-risk patients. In some patients, doses were repeated at the discretion of the investigator performing the challenges.

| DOSES | Peanut protein (g) |
| --- | --- |
| 1* | 0.033* |
| 2 | 0.1 |
| 3 | 0.25 |
| 4 | 0.5 |
| 5 | 1.0 |
| 6 | 2.5 |
| 7 | 5.0 |

## Table E4. Criteria for positive oral food challenge to peanut. A positive oral food challenge (OFC) was defined by the presence of either ≥1 major criteria or ≥2 minor criteria. An indeterminate OFC was defined as one minor criterion. A negative OFC was defined by the absence of major or minor criteria.

| Major criteria | Confluent erythematous pruritic rash |
| --- | --- |
|  | Wheezing |
|  | Stridor |
|  | Dysphonia / Aphonia |
|  | ≥ 3 urticarial lesions |
|  | ≥ 1 site of angioedema |
|  | Hypotension for age not related to vasovagal episode |
|  | Evidence of severe abdominal pain that persists for ≥3 minutes |
| Minor criteria | Vomiting |
|  | Diarrhoea |
|  | Persistent rubbing of eyes that last ≥3 minutes |
|  | Persistent rhinorrhea that lasts ≥3 minutes |
|  | Persistent scratching that lasts ≥3 minutes |

## Table E5. Grading systems used to classify the severity of allergic reactions during oral food challenges^4, 5^. If criteria for more than one grade applied, the highest grade was considered to classify the allergic reaction.

Common Terminology Criteria for Adverse Events (CTCAE) Severity AE Grading:

Adverse events were graded on a scale from 1 to 5 according to the following standards in the NCI‑CTCAE manual:

Grade 1 = mild adverse event (transient flushing or rash, not requiring treatment).

Grade 2 = moderate adverse event (required treatment and responded promptly to symptomatic treatment).

Grade 3 = severe and undesirable adverse event (prolonged response to treatment or recurrence of symptoms following initial improvement or hospitalization required).

Grade 4 = life-threatening or disabling adverse event (change in the level of consciousness or vital signs that can be life-threatening and require urgent treatment).

Grade 5 = death.

Ewan & Clark

Grade 1 (mild)—localized cutaneous erythema/ urticaria/angioedema/oral pruritis

Grade 2 (mild)—generalized erythema/urticaria/angio-oedema

Grade 3 at least grade 1 or 2 plus gastrointestinal symptoms/rhinoconjunctivitis

Grade 4 (moderate): mild laryngeal oedema (voice change/tightening of throat)/mild asthma

Grade 5 (severe): pronounced dyspnoea/hypotensive symptoms (light-headedness/ collapse/ loss of consciousness)

Categorical Designations based on drugs administered during allergic reaction.

Mild: antihistamines (anti-H1 and/or anti-H2) and/or prednisolone/hydrocortisone

Moderate: salbutamol, O2

Severe: epinephrine, iv saline

Drug Severity Point System

1 antihistamines (anti-H1 and/or anti-H2) and/or prednisolone/hydrocortisone and/or O2

2 salbutamol

3 epinephrine or iv saline

4 epinephrine and iv saline

5 epinephrine >1x

**Table E6.** Skin prick test to peanut, specific IgE and IgG4 levels to peanut or peanut allergens (as indicated) and basophil activation test to peanut in peanut avoidance and peanut consumption groups and in peanut allergic and non-allergic subjects in the LEAP, LEAP-On and PAS studies.

|  |  | **Peanut Avoidance** | | **Peanut Consumption** | | **Peanut Allergic** | | **Not Peanut Allergic** | |
| --- | --- | --- | --- | --- | --- | --- | --- | --- | --- |
| **Study** | **Allergy Tests** | **N** | **Mean (SD)** | **N** | **Mean (SD)** | **N** | **Mean (SD)** | **N** | **Mean (SD)** |
| LEAP | SPT (mm) | 252 | 2.1 (4.2) | 222 | 0.9 (2.7) | 54 | 10.0 (5.0) | 418 | 0.4 (1.1) |
|  | P-sIgE (KU/L) | 252 | 8.3 (35.8) | 222 | 2.8 (11.7) | 54 | 41.9 (71.1) | 418 | 1.1 (4.5) |
|  | Ara h 1-sIgE (KU/L) | 250 | 3.2 (22.0) | 219 | 0.5 (3.6) | 52 | 17.0 (46.6) | 415 | 0.1 (0.5) |
|  | Ara h 2-sIgE (KU/L) | 250 | 7.0 (37.5) | 219 | 0.7 (6.3) | 52 | 36.2 (77.1) | 415 | 0.0 (0.2) |
|  | Ara h 3-sIgE (KU/L) | 250 | 0.9 (7.2) | 219 | 0.3 (1.6) | 52 | 4.5 (15.5) | 415 | 0.1 (1.1) |
|  | Ara h 8-sIgE (KU/L) | 250 | 1.4 (8.0) | 218 | 1.8 (9.1) | 52 | 5.4 (19.5) | 414 | 1.1 (5.7) |
|  | Ara h 9-sIgE (KU/L) | 250 | 0.2 (1.3) | 219 | 0.5 (4.1) | 52 | 0.7 (2.9) | 415 | 0.3 (3.0) |
|  | P-sIgG4 (KU/L) | 251 | 631.2 (2135.1) | 221 | 2454.8 (5743.2) | 53 | 1252.1 (3074.8) | 417 | 1521.2 (4463.6) |
|  | IgG4/IgE Ratio (log-10) | 251 | 2.3 (0.5) | 221 | 2.9 (0.7) | 53 | 2.5 (0.6) | 417 | 2.5 (0.7) |
|  | BAT to peanut (% CD63+ basophils) | 252 | 5.7 (16.9) | 222 | 1.6 (7.4) | 54 | 29.7 (28.5) | 418 | 0.3 (1.1) |
| LEAP-On | SPT (mm) | 219 | 2.3 (4.6) | 203 | 1.1 (2.6) | 51 | 10.4 (4.4) | 371 | 0.5 (1.3) |
|  | P-sIgE (KU/L) | 219 | 9.6 (54.7) | 202 | 3.3 (11.5) | 51 | 45.0 (108.3) | 369 | 1.3 (5.3) |
|  | Ara h 1-sIgE (KU/L) | 215 | 1.7 (9.7) | 200 | 0.5 (3.4) | 50 | 8.9 (19.7) | 364 | 0.1 (0.2) |
|  | Ara h 2-sIgE (KU/L) | 215 | 3.7 (18.7) | 200 | 0.7 (5.3) | 50 | 18.4 (36.9) | 364 | 0.1 (0.3) |
|  | Ara h 3-sIgE (KU/L) | 215 | 0.8 (6.5) | 200 | 0.3 (1.2) | 50 | 3.7 (13.3) | 364 | 0.1 (0.8) |
|  | Ara h 8-sIgE (KU/L) | 215 | 2.8 (12.5) | 199 | 2.3 (9.2) | 50 | 6.7 (18.7) | 363 | 2.0 (9.4) |
|  | Ara h 9-sIgE (KU/L) | 215 | 0.3 (2.4) | 198 | 0.3 (2.1) | 50 | 1.1 (5.0) | 362 | 0.2 (1.5) |
|  | P-sIgG4 (KU/L) | 219 | 667.1 (1922.7) | 201 | 1652.8 (6528.7) | 51 | 1422.7 (2836.3) | 368 | 1102.4 (4958.9) |
|  | IgG4/IgE Ratio (log-10) | 219 | 2.2 (0.6) | 201 | 2.6 (0.6) | 51 | 2.5 (0.7) | 368 | 2.4 (0.6) |
|  | BAT to peanut (% CD63+ basophils) | 219 | 4.7 (14.9) | 204 | 1.8 (7.0) | 51 | 23.7 (25.7) | 371 | 0.5 (2.3) |
| PAS | SPT (mm) |  |  |  |  | 33 | 10.4 (3.1) | 46 | 0.2 (0.9) |
|  | P-sIgE (KU/L) |  |  |  |  | 33 | 45.8 (67.6) | 46 | 0.2 (0.6) |
|  | Ara h 1-sIgE (KU/L) |  |  |  |  | 27 | 7.0 (15.3) | 45 | 0.0 (0.1) |
|  | Ara h 2-sIgE (KU/L) |  |  |  |  | 27 | 22.3 (35.0) | 45 | 0.0 (0.1) |
|  | Ara h 3-sIgE (KU/L) |  |  |  |  | 26 | 0.9 (3.0) | 45 | 0.0 (0.1) |
|  | Ara h 8-sIgE (KU/L) |  |  |  |  | 27 | 1.4 (2.4) | 45 | 1.4 (9.3) |
|  | Ara h 9-sIgE (KU/L) |  |  |  |  | 26 | 0.1 (0.1) | 45 | 0.0 (0.0) |
|  | P-sIgG4 (KU/L) |  |  |  |  | 33 | 774.5 (2262.8) | 46 | 372.6 (703.0) |
|  | IgG4/IgE Ratio (log-10) |  |  |  |  | 33 | 2.4 (0.5) | 46 | 2.2 (0.5) |
|  | BAT to peanut (% CD63+ basophils) |  |  |  |  | 33 | 34.5 (26.8) | 46 | 0.2 (0.5) |

**Table E7.** Comparison of clinical characteristics between participants with and without BAT measurements among LEAP, LEAP-On and PAS participants.

|  | **LEAP** | | | **LEAP-On** | | | **PAS** | | | |
| --- | --- | --- | --- | --- | --- | --- | --- | --- | --- | --- |
|  | **BAT Not Performed** | **BAT Performed** | **P** | **BAT Not Performed** | **BAT Performed** | **P** | **BAT Not Performed** | **BAT Performed** | **P** | |
|  | **(N = 86)** | **(N = 335)** |  | **(N = 118)** | **(N = 266)** |  | **(N = 37)** | **(N = 76)** |  | |
| Allergic | 5 (6%) | 59/333 (18%) | 0.006 | 12/112 (11%) | 53 (20%) | 0.03 | 3/24 (12%) | 37/71 (52%) | 0.001 | |
| Male Gender | 59 (69%) | 211 (63%) | 0.332 | 75 (64%) | 172 (65%) | 0.835 | 23 (62%) | 51 (67%) | 0.604 | |
| Age | 67.8 (1.6) | 67.8 (1.8) | 0.973 | 80 (1.7) | 79.7 (1.8) | 0.209 | 68.1 (2) | 67.9 (1.8) | 0.517 | |
| Eczema | 42 (49%) | 141 (42%) | 0.26 | 47/113 (42%) | 108 (41%) | 0.857 | 31/35 (89%) | 72/74 (97%) | 0.062 | |
| Ewan & Clark Severe | 0/4 (0%) | 14/53 (26%) | 0.237 | 2/5 (40%) | 2/31 (6%) | 0.027 | 1/3 (33%) | 8/36 (22%) | 0.661 | |
| CTCAE Severe | 0/4 (0%) | 7/53 (13%) | 0.438 | 0/5 (0%) | 1/31 (3%) | 0.684 | 0/3 (0%) | 5/36 (14%) | 0.489 | |
| Medication Severe | 0/4 (0%) | 9/53 (17%) | 0.369 | 2/4 (50%) | 1/31 (3%) | 0.002 | 0/3 (0%) | 7/36 (19%) | 0.399 | |
| Dose Tolerated (corrected) | 6.3 (2.4) (N=84) | 7.5 (3.3) (N=330) | < 0.001 | 7.5 (2.5) (N=104) | 8 (2.8) (N=246) | 0.029 | 7.7 (3.2)  (N=24) | 4.5 (4.5)  (N=73) | 0.002 | |
| Dose Tolerated | 6.3 (2.4) (N=84) | 6.4 (3.2) (N=330) | 0.436 | 6.3 (2.4) (N=104) | 7.1 (2.9) (N=246) | 0.004 | 4.6 (1.9)  (N=24) | 3.2 (3.4)  (N=73) | 0.015 | |
| Positive OFC | 4 (5%) | 53 (16%) | 0.007 | 5 (4%) | 31 (12%) | 0.021 | 3 (8%) | 36 (47%) | < 0.001 | |
| *Note:* | | | | | | | | | |  |
| Summaries presented as N (%) or mean (sd). | | | | | | | | | |  |
| Continuous variables compared using Wilcoxon rank-sum test, categorical variables compared using Pearson’s chi-squared test. | | | | | | | | | |  |

**Table E8.** Comparison of biomarkers between participants with and without BAT measurements among LEAP, LEAP-On and PAS participants eligible for BAT.

|  | **LEAP** | | | **LEAP-On** | | | **PAS** | | |
| --- | --- | --- | --- | --- | --- | --- | --- | --- | --- |
|  | **BAT Not Performed (N = 86)** | **BAT Performed (N = 335)** | **P** | **BAT Not Performed (N = 118)** | **BAT Performed (N = 266)** | **P** | **BAT Not Performed (N = 37)** | **BAT Performed (N = 76)** | **P** |
| Arah1-sIgE (KU/L) | 0.05 (0.09)  (N=83) | 2.81 (19.48)  (N=326) | 0.539 | 1.25 (10.38)  (N=89) | 1.81 (9.25)  (N=259) | < 0.001 | 13.11 (36.89)  (N=23) | 5.16 (16.58)  (N=67) | 0.044 |
| Arah2-sIgE (KU/L) | 0.44 (2.44)  (N=83) | 5.84 (33.30)  (N=326) | 0.234 | 2.24 (14.47)  (N=88) | 3.62 (17.62)  (N=259) | < 0.001 | 17.81 (56.09)  (N=23) | 17.54 (54.26)  (N=67) | 0.025 |
| Arah3-sIgE (KU/L) | 0.05 (0.11)  (N=83) | 0.90 (6.45)  (N=326) | 0.476 | 0.16 (0.74)  (N=88) | 0.89 (6.04)  (N=259) | < 0.001 | 1.72 (5.56)  (N=23) | 1.30 (7.77)  (N=66) | 0.175 |
| Arah8-sIgE (KU/L) | 1.33 (4.26)  (N=83) | 2.43 (10.50)  (N=325) | 0.385 | 2.82 (9.46)  (N=88) | 4.20 (13.76)  (N=258) | 0.002 | 0.49 (2.04)  (N=23) | 1.64 (7.76)  (N=67) | 0.099 |
| Arah9-sIgE (KU/L) | 0.03 (0.05)  (N=83) | 0.46 (3.55)  (N=326) | 0.555 | 0.04 (0.08)  (N=88) | 0.46 (2.87)  (N=257) | 0.001 | 0.02 (0.02)  (N=23) | 0.04 (0.10)  (N=66) | 0.457 |
| IgG4/IgE ratio (log) | 2.73 (0.83)  (N=84) | 2.80 (1.08)  (N=334) | 0.283 | 2.74 (1.07)  (N=98) | 2.57 (1.06)  (N=265) | 0.176 | 2.75 (1.25)  (N=26) | 2.00 (1.35) | 0.006 |
| Peanut sIgG4 (micrograms/L) | 1388.10 (2005.08)  (N=84) | 2058.83 (5109.42)  (N=334) | 0.667 | 900.71 (2350.56)  (N=98) | 1625.70 (5905.16)  (N=265) | 0.095 | 321.54 (434.09)  (N=26) | 722.11 (1741.86) | 0.043 |
| BAT to peanut (% basophils to 10-100 ng/ml peanut) (imputed) | 0.00 (0.00)  (N=9) | 5.41 (15.73) | 0.022 | 0.00 (0.00)  (N=38) | 5.24 (14.62) | < 0.001 | 0.00 (0.00)  (N=18) | 19.12 (27.41) | < 0.001 |
| BAT to peanut (% basophils to 10-100 ng/ml peanut) | (N=0) | 5.41 (15.73) |  | (N=0) | 5.24 (14.62) |  | (N=0) | 19.12 (27.41) |  |
| Peanut IgE (KU/L) | 1.51 (4.99)  (N=85) | 8.25 (32.35) | 0.143 | 7.27 (35.32)  (N=98) | 10.49 (50.43)  (N=265) | 0.014 | 23.96 (77.41)  (N=26) | 30.83 (68.95) | 0.002 |
| Peanut SPT (mm) | 1.10 (2.78) | 2.31 (4.28)  (N=334) | 0.02 | 1.33 (3.37)  (N=110) | 2.79 (4.51) | < 0.001 | 3.42 (6.02)  (N=31) | 5.93 (5.53) | 0.015 |
| *Note:* | | | | | | | | | |
| Mean (SD) | | | | | | | | | |
| Comparisons use Wilcoxon rank-sum test | | | | | | | | | |

**Table E9.** Comparison of the various biomarkers between severe reactors and non-severe reactors using Wilcoxon test.

| **Biomarkers** | **N** | **Not Severe**  **(N = 532)** | **Severe**  **(N = 12)** | **Wilcoxon P-value** |
| --- | --- | --- | --- | --- |
| %CD63+ Basophils | 544 | 0 [0, 0.78] | 58.4 [52.9, 76.4] | P < 0.001 |
| Peanut SPT | 544 | 0 [0, 0] | 10 [8.8, 13.3] | P < 0.001 |
| rAra h 2-specific IgE | 533 | 0.01[0.01, 0.03] | 60.9 [17.5, 114.5] | P < 0.001 |
| Peanut-specific IgE | 544 | 0.04 [0.01, 0.5] | 98.0 [34.1, 191.8] | P < 0.001 |
| Peanut-specific IgG4 | 542 | 220 [70, 988] | 295 [155, 652] | P = 0.55 |
| IgG4/IgE ratio | 542 | 2256 [415, 5177] | 1.5 [0.7, 9.9] | P < 0.001 |

**Table E10.** Optimal cut-offs to classify subjects at high-risk for severe allergic reactions during the oral peanut challenges according to the Ewan and Clark and the Medication Grading severity scales. Optimal cut-offs were determined based on the Youden’s index which is the distance between the point of inflexion of the ROC curve and the reference line. Sensitivity, specificity, positive predictive value (PPV) and negative predictive value (NPV) with 95% confidence intervals are indicated for each cut-off.

| Biomarker | Type | BAT  (%CD63+ Basophils) | Ara h 2-sIgE (KU/L) | Peanut-sIgE (KU/L) | Peanut-sIgG4 (μg/L) | IgG4/IgE Ratio | SPT Peanut  (mm) |
| --- | --- | --- | --- | --- | --- | --- | --- |
| Ewan and Clark  grading | Threshold | 10 | 0.2 | 1.3 | 375 | 2.1 | 5.5 |
|  | Sensitivity | 95 (91 ­ 100) | 95 (82 ­ 100) | 95 (82 ­ 100) | 59 (14 ­ 91) | 95 (86 ­ 100) | 100 (100 ­ 100) |
|  | Specificity | 94 (82 ­ 96) | 82 (71 ­ 97) | 82 (71 ­ 97) | 58 (32 ­ 98) | 85 (77 ­ 94) | 90 (87 ­ 93) |
|  | PPV | 40 (19 ­ 53) | 18 (13 ­ 58) | 18 (13 ­ 58) | 6 (5 ­ 21) | 21 (15 ­ 40) | 29 (24 ­ 37) |
|  | NPV | 100 (100 ­ 100) | 100 (100 ­ 100) | 100 (99 ­ 100) | 97 (96 ­ 99) | 100 (99 ­ 100) | 100 (100 ­ 100) |
| Medication grading | Threshold | 10 | 1.4 | 3.1 | 175 | 2.5 | 7.5 |
|  | Sensitivity | 100 (100 ­ 100) | 100 (100 ­ 100) | 100 (94 ­ 100) | 81 (25 ­ 100) | 100 (88 ­ 100) | 100 (100 ­ 100) |
|  | Specificity | 93 (91 ­ 98) | 94 (92 ­97) | 88 (85 ­ 97) | 46 (32 ­ 94) | 78 (75 ­ 93) | 92 (90 ­ 95) |
|  | PPV | 31 (26 ­ 59) | 30 (25 ­ 52) | 20 (17­ 48) | 4 (4 ­ 14) | 12 (11 ­ 30) | 28 (23 ­ 36) |
|  | NPV | 100 (100 ­ 100) | 100 (100 ­ 100) | 100 (100 ­ 100) | 99 (98 ­ 100) | 100 (100 ­ 100) | 100 (100 ­ 100) |

**Table E11.** Multivariable analyses, with terms entered into model as 2 degrees-of-freedom flexible restricted cubic splines. Tests of parameters from proportional odds logistic regression model of reaction severity describe the statistical significance of the groups of terms related to each of the three predictors in the model, where each predictor is entered into the model as a flexible restricted cubic spline represented by two model parameters. For example, the significance test for the two terms associated with BAT (a linear and a non-linear term) shows that BAT is significantly associated with reaction severity after adjusting for Ara h 2 and peanut SPT (P = 0.002).

| **Term** | **Test Statistic** | **P-value** |
| --- | --- | --- |
| Ara h 2 | $\chi_{2}^{2}=1.15$ | 0.563 |
| Peanut SPT | $\chi_{2}^{2}=68.2$ | < 0.001 |
| BAT | $\chi_{2}^{2}=12.8$ | 0.002 |

# Table E12. External validation of severity and dose threshold models. The tables below shows external validation of models of (A) severity and (B) dose threshold within the validation set, ordered by the discrimination index (C-index). Broadly speaking, model discrimination within this set is not as good, and errors are much higher. Notably, IgE and variables involving IgE seem to be related to risk in the opposite direction to that seen in the original model. This is potentially one explanation for why the multivariable models do not seem to perform as well as they did in the internal validation.

1. **Severity**

|  |  | **Absolute calibration error** | |
| --- | --- | --- | --- |
|  |  | **Moderate or higher** | |
| **Model** | **C-Index** | **Mean** | **90th %ile** |
| Multivariable (BAT) | 1.00 | 22.8 | 85.3 |
| Multivariable (No BAT) | 1.00 | 21.5 | 83.0 |
| SPT | 1.00 | 28.0 | 90.3 |
| Ara h 2-specific IgE | 0.85 | 45.3 | 99.5 |
| BAT | 0.98 | 22.1 | 67.4 |
| IgE to peanut | 1.00 | 38.9 | 94.1 |
| IgG4 to peanut | 0.76 | 35.2 | 58.2 |

1. **Threshold**

|  |  | **Absolute Calibration Error** | | | | | |
| --- | --- | --- | --- | --- | --- | --- | --- |
|  |  | **0.1 g** | | **5.0 g** | | **9.35 g** | |
|  | **C-index** | **Mean** | **90th %ile** | **Mean** | **90th %ile** | **Mean** | **90th %ile** |
| Net % CD63+ | 0.869 | 6 | 22.4 | 11.5 | 35.2 | 7.8 | 24.6 |
| Multivariable | 0.738 | 3.2 | 5.6 | 12.4 | 43.9 | 10.8 | 35.6 |
| Wheal | 0.679 | 10.1 | 29.3 | 21.2 | 67.7 | 22.8 | 64 |
| Multivariable (no BAT) | 0.667 | 6.3 | 13.3 | 20.5 | 58.5 | 21.6 | 55.1 |
| Ara h2 | 0.571 | 3.8 | 3.9 | 7.4 | 7.9 | 11.7 | 12.5 |
| IgG4 | 0.539 | 1.6 | 3 | 1.6 | 2.3 | 5.1 | 6.7 |
| log10(Igg4/IgE) | 0.341 | 17.7 | 29.5 | 32.4 | 52.9 | 33.3 | 52.1 |
| IgE | 0.304 | 12.8 | 28 | 25.5 | 49.8 | 28.7 | 49.9 |

**E-References:**

1. Du Toit G, Roberts G, Sayre PH, Plaut M, Bahnson HT, Mitchell H, et al. Identifying infants at high risk of peanut allergy: the Learning Early About Peanut Allergy (LEAP) screening study. J Allergy Clin Immunol 2013; 131:135-43 e1-12.

2. Du Toit G, Roberts G, Sayre PH, Bahnson HT, Radulovic S, Santos AF, et al. Randomized trial of peanut consumption in infants at risk for peanut allergy. N Engl J Med 2015; 372:803-13.

3. Du Toit G, Sayre PH, Roberts G, Sever ML, Lawson K, Bahnson HT, et al. Effect of Avoidance on Peanut Allergy after Early Peanut Consumption. N Engl J Med 2016; 374:1435-43.

4. Ewan PW, Clark AT. Long-term prospective observational study of patients with peanut and nut allergy after participation in a management plan. Lancet 2001; 357:111-5.

5. Available from https://evs.nci.nih.gov/ftp1/CTCAE/About.html.

**E-Figure legends:**

**Figure E1.** Logistic growth function used to compute CD-sens. M represents the concentration at which half of the maximum activation value is attained. CD-sens is then computed directly from this value as the antilogarithm in base 10 of M.

**Figure E2.** Agreement between the severity scales used to grade the allergic reactions during the peanut challenges in the LEAP, LEAP-On and PAS studies measured with Kendall’s Rank Correlation (Kendall’s tau = 0.12 to 0.64).

**Figure E3.** Basophil activation (measured as %CD63+ basophils corrected for spontaneous activation, i.e. minus %CD63+ basophils in the absence of *in vitro* stimulation) to increasing concentrations of peanut extract in LEAP, LEAP-On, and PAS study participants. BAT and allergic status were determined at approximately age 5 in LEAP and PAS studies and at approximately age 6 in the LEAP-On study. Each line represents a smoothed dose response curve for each individual, and the colours distinguish the different combinations of allergic status and reaction severity.

**Figure E4.** Basophil activation (measured as %CD63+ basophils corrected for spontaneous activation, i.e. minus %CD63+ basophils prior to stimulation) to the positive controls in peanut allergic and non-allergic participants in the PAS, LEAP and LEAP-On studies. Basophil activation in response to anti-IgE, anti-FceRI and fMLP was similar across allergic status; however, the basophil response to anti-IgE tended to be higher in peanut allergic compared to non-allergic children (median 46.7 vs 36.8, p=0.094). The basophil activation in response to fMLP was similar between severe (n=13) and non-severe OFC reactions (Median [IQR] 49.1 [36.6 – 63.3] and 43.8 [28.3 – 59.2], respectively; P=0.30), while basophil activation was higher for severe OFC reactions both in response to anti-FceRI (28.6 [13.4 – 46.7] vs. 14.6 [6.4 – 24.8], P = 0.026) and anti-IgE (Median [IQR] 61.7 [49.1 – 72.2] vs. 37.0 [15.5 – 56.6], P = 0.001).

**Figure E5A.** Results of skin prick test for peanut allergic versus non-allergic participants in the LEAP and PAS studies. Results for children with severe or life-threatening allergic reactions are indicated in red (7 in LEAP and 5 in PAS). p values refer to the comparison between allergic and non-allergic participants.

**Figure E5B.** Results of Ara h 2-specific IgE in kilounits per liter for peanut allergic versus non-allergic participants in the LEAP and PAS studies. Results for children with severe or life-threatening allergic reactions are indicated in red (7 in LEAP and 5 in PAS). p values refer to the comparison between allergic and non-allergic participants.

**Figure E5C.** Results of peanut-specific IgE in kilounits per liter for peanut allergic versus non-allergic participants in the LEAP and PAS studies. Results for children with severe or life-threatening allergic reactions are indicated in red (7 in LEAP and 5 in PAS). p values refer to the comparison between allergic and non-allergic participants.

**Figure E5D.** Results of peanut-specific IgG4 in micrograms per liter for peanut allergic versus non-allergic participants in the LEAP and PAS studies. Results for children with severe or life-threatening allergic reactions are indicated in red (7 in LEAP and 5 in PAS). p values refer to the comparison between allergic and non-allergic participants.

**Figure E5E.** Results of IgG4/IgE ratios for peanut allergic versus non-allergic participants in the LEAP and PAS studies. Results for children with severe or life-threatening allergic reactions are indicated in red (7 in LEAP and 5 in PAS). p values refer to the comparison between allergic and non-allergic participants.

**Figure E6.** Basophil activation (measured as %CD63+ basophils corrected for spontaneous activation, i.e. minus %CD63+ basophils prior to stimulation) to peanut in participants in the PAS and LEAP studies with lower (<0.1g) or higher (≥0.1g) cumulative threshold dose of peanut protein.

**Figure E7.** Survival curve showing estimated proportion of participants who remain reaction-free as a function of cumulative peanut dose, for BAT values of 0, 5, 10, 20, and 70 can be used to predict the risk of having an allergic reaction to low dose of peanut from the result of BAT. For example, from this plot we might expect for participants with BAT values of 10 that about 40% will tolerate about 1g of peanut before having a reaction during OFC, and about 20% will tolerate about 6g. This contrasts with those having a BAT of 5 of whom 80% and 70% will tolerate 1g and 6g, respectively.

**Figure E8.** Scatterplot of CD-sens by total tolerated dose of peanut during OFC, showing generally lower CD-sens values for higher tolerated peanut doses (Spearman’s rho = -0.3, P < 0.001). CD-sens has been plotted on the log_10_-scale.

## Figure E9. Basophil activation (measured as %CD63+ basophils corrected for spontaneous activation, i.e. minus %CD63+ basophils prior to stimulation) to peanut in the LEAP and PAS studies' participants with different severity scores and cumulative threshold dose during the peanut challenges. Participants whose severity was not graded included both participants who did not undergo or had a negative peanut challenge. Peanut allergic participants are marked in red.

# Figure E10. Additional nomograms for severity using different combinations of tests.

1. Nomogram for severity of allergic reactions to peanut using SPT, BAT and Ara h 2-specific IgE.
2. Nomogram for severity of allergic reactions to peanut using SPT and BAT.
3. Nomogram for severity of allergic reactions to peanut using SPT and Ara h 2-specific IgE.
4. Nomogram for severity of allergic reactions to peanut using SPT.
5. Nomogram for severity of allergic reactions to peanut using BAT and Ara h 2-specific IgE.
6. Nomogram for severity of allergic reactions to peanut using BAT.
7. Nomogram for severity of allergic reactions to peanut using Ara h 2-specific IgE.
8. Nomogram for severity of allergic reactions to peanut using SPT and peanut-specific IgE.

**Figure E11.** Nomogram for dose threshold model using Ara h 2-specific IgE, skin prick test (Pnt Wheal) and peanut-specific IgG4/IgE ratios.

**Figure E12.** Parallel coordinates plot showing CTCAE reaction severity category along the horizontal axis, and the model-based predicted probabilities of being in each of these categories along the vertical axis; each line represents the model predicted probabilities for a given participant and add to 100% across the outcomes. Subjects are panelled by observed CTCAE reaction severity outcome. The colours represent cases where the model correctly predicted the outcome (blue) and where the model gave a discordant result (red). Concordance and discordance was defined at the 50% probability threshold. Overall, the concordance rate was 96% (906/945).

**Figure E13.** Calibration plots for assessing accuracies for predicting probabilities defining model having a moderate or more severe reaction, and having Severe/Life-threatening reactions, as assessed by the CTCAE grading scale.

## Figure E13A. Model with BAT

**Figure E13B.** Model without BAT

**Figure E14A.** Calibration curve for predictions of moderate or more severe reactions based on CTCAE score from the model with BAT within external validation set. Note that within this validation dataset the only reactions seen were of the moderate level.

**Figure E14B.** Calibration curve for predictions of moderate or more severe reactions based on CTCAE score from the model without BAT within external validation se
